# Effects of metal amendment and metalloid supplementation on foliar defences are plant accession-specific in the hyperaccumulator *Arabidopsis halleri*

**DOI:** 10.1007/s10534-023-00550-5

**Published:** 2023-10-24

**Authors:** Rocky Putra, Max Tölle, Ute Krämer, Caroline Müller

**Affiliations:** 1 Department of Chemical Ecology, Bielefeld University, Universitätsstr. 25, 33615 Bielefeld, Germany; 2 Department of Molecular Genetics and Physiology of Plants, Ruhr University Bochum, Universitätsstr. 150, 44801 Bochum, Germany

**Keywords:** Elemental defence, Glucosinolate, Intraspecific variation, Metal hyperaccumulation, Silicon, Trade-off hypothesis

## Abstract

Soil pollution by metals and metalloids as a consequence of anthropogenic industrialisation exerts a seriously damaging impact on ecosystems. However, certain plant species, termed hyperaccumulators, are able to accumulate extraordinarily high concentrations of these metal(loid)s in their aboveground tissues. Such hyperaccumulation of metal(loid)s is known to act as a defence against various antagonists, such as herbivores and pathogens. We investigated the influences of metal(loid)s on potential defence traits, such as foliar elemental, organic and mechanical defences, in the hyperaccumulator plant species *Arabidopsis halleri* (Brassicaceae) by artificially amending the soil with common metallic pollutants, namely cadmium (Cd) and zinc (Zn). Additionally, unamended and metal-amended soils were supplemented with the metalloid silicon (Si) to study whether Si could alleviate metal excess. Individuals originating from one non-/low- and two moderately to highly metal-contaminated sites with different metal concentrations (hereafter called *accessions*) were grown for eight weeks in a full-factorial design under standardised conditions. There were significant interactive effects of metal amendment and Si supplementation on foliar concentrations of certain elements (Zn, Si, aluminium (Al), iron (Fe), potassium (K) and sulfur (S), but these were accession-specific. Profiles of glucosinolates, characteristic organic defences of Brassicaceae, were distinct among accessions, and the composition was affected by soil metal amendment. Moreover, plants grown on metal-amended soil contained lower concentrations of total glucosinolates in one of the accessions, which suggests a potential trade-off between inorganic defence acquisition and biosynthesis of organic defence. The density of foliar trichomes, as a proxy for the first layer of mechanical defence, was also influenced by metal amendment and/or Si supplementation in an accession-dependent manner. Our study highlights the importance of examining the effects of co-occurring metal(loid)s in soil on various foliar defence traits in different accessions of a hyperaccumulating species.

**Supplementary Information:**

The online version contains supplementary material available at 10.1007/s10534-023-00550-5.

## Introduction

Soil metal(loid) pollution particularly due to human industrial and mining activities is a serious concern that affects various environmental, health and socio-economic aspects worldwide (FAO and UNEP 2021). Arsenic (As), cadmium (Cd), mercury (Hg), lead (Pb), nickel (Ni) and zinc (Zn) are known to be the predominant metal(loid) contaminants in soils. However, certain plant species, known as hyperaccumulators, exhibit the capacity to accumulate extraordinary levels of one, and sometimes several, metal(loid)s in aboveground tissues (Krämer 2010). Such plants may be used to remediate metal(loid)-contaminated sites and mitigate the far-reaching adverse consequences of soil pollutant metal(loid)s for the environment (Rylott and Bruce 2022). Fundamental studies on the combined effects of different metal(loid)s, which often co-occur in soils, on hyperaccumulators are still lacking (Putra and Müller 2023). Moreover, responses to co-occurring metal(loid)s may differ between populations within the same species. Yet, little is known about such differences (but see Kazemi-Dinan et al. 2015b; Stein et al. 2017) and whether they are linked to plant defence types, which could offer insights in their ecological functions in terrestrial ecosystems.

About 700 plant species are known so far that can hyperaccumulate certain metal(loid)s, mostly belonging to the Brassicaceae and Phyllanthaceae (Reeves et al. 2018). The main criterion to identify a given plant taxon as a hyperaccumulator is the metal(loid) concentration accumulated in any type of aboveground organ (e.g. leaves). For example, threshold criteria for Cd and Zn hyperaccumulation are > 100 and > 3000 µg/g dry biomass, respectively (Reeves et al. 2018). Some hyperaccumulators are able to accumulate more than one metal/metalloid. For example, *Arabidopsis halleri* (Brassicaceae) is known to hyperaccumulate Cd, Zn and Pb (Stein et al. 2017). Members of certain plant families, such as the Poaceae (e.g. *Oryza sativa*), Asteraceae (e.g. *Helianthus annuus*) and Fabaceae (e.g. *Cajanus cajan*), can accumulate the metalloid silicon (Si) up to 10% on a dry biomass basis (Putra et al. 2020). Si is the most ubiquitous metalloid in soils (Epstein 1999). This metalloid can alleviate metal stress in plants (Meena et al. 2021), but its potential involvement in metal hyperaccumulation is poorly understood. However, recent studies demonstrated that Si supplementation seemed to alleviate metal(loid) excess, for example, by lowering As and Cd concentrations either in roots or shoots of an As hyperaccumulator, *Isatis cappadocica* (Brassicaceae) (Azam et al. 2021) or by (indirectly) stimulating shoot growth of the lanthanum (La) hyperaccumulator *Dicranopteris linearis* (Zheng et al. 2023), relative to Si-unsupplemented plants. Hyperaccumulation of metal(loid)s is usually accompanied by hypertolerance. Therefore, metal(loid) hyperaccumulation often causes no apparent toxicity symptoms in these plants, probably as a result of specific evolutionary adaptations allowing physiological adjustment of an ion homeostasis at the cellular level (Clemens 2001; Krämer 2010). Moreover, it is presumed that metal(loid) hyperaccumulation can affect foliar concentrations of other elements beyond the metals that are hyperaccumulated. This is often the case for other elements, such as iron (Fe), potassium (K), manganese (Mn) and sulfur (S), which may play an important role in metal tolerance and detoxification (Clemens 2001; Andresen et al. 2018). Numerous studies provided evidence supporting that metal(loid) hyperaccumulation confers an effective elemental defence against a broad range of antagonists, such as herbivorous insects and microbial pathogens, which has been termed the elemental defence hypothesis (Boyd 2007). For example, Cd and Zn hyperaccumulation in *A. halleri* increases plant resistance against various leaf-chewing and phloem-sucking insects (Kazemi-Dinan et al. 2014; Stolpe et al. 2017). Likewise, high Si accumulation in the foliage and in trichomes resulted in a potent mechanical defence in *O. sativa* against folivores (Andama et al. 2020). In addition to elemental and mechanical defences acquired through metal(loid) hyperaccumulation, hyperaccumulators also contain numerous specialised metabolites that can act as organic defences against antagonists (Putra and Müller 2023). The biosynthesis of specialised metabolites is considered to be more costly than metal(loid) hyperaccumulation (Boyd 2013). Thus, studying how hyperaccumulators fine-tune these two types of defences may offer insights into potential trade-offs.

Glucosinolates are characteristic specialised metabolites of the Brassicales species. These metabolites and particularly their hydrolysis products, which are formed after tissue damage, are well-documented for their defensive functions against herbivores and pathogens (Hopkins et al. 2009). Glucosinolates consist of an S-*β*-d-glucopyrano unit linked to an O-sulfated (*Z*)-thiohydroximate function (Fig. 4). They can be broadly grouped according to the amino acid precursor of their side chain into aliphatic, benzenic and indole glucosinolates, whereby aliphatic glucosinolates are structurally most diverse (Blažević et al. 2020). Several studies revealed that metal amendment of the growth substrate with Cd and/or Zn alters foliar concentrations of glucosinolates in Brassicales hyperaccumulators, such as *Noccaea* (formerly *Thlaspi*) *praecox* (Tolrà et al. 2006), *N.* *caerulescens* (Tolrà et al. 2001) and *A*. *halleri* (Stolpe et al. 2017). Hyperaccumulation of metals, such as Zn and Cd, and the biosynthesis of glucosinolates may be metabolically intertwined due to the involvement of S metabolism (Ernst et al. 2008; Pongrac et al. 2010). Therefore, trade-offs (Boyd 2013) may be expected in the investment of both types of defences, but also joint effects (Boyd 2012), between but also within species.

The Zn and facultative Cd hyperaccumulator *A. halleri* is widely distributed across Asia and Europe and is a diploid outcrossing perennial that shows clonal growth (Honjo and Kudoh 2019). It serves as a model organism in cross-species comparative approaches within the *Arabidopsis* genus and in studies of population differentiation, including local adaptation to soil metal contamination (Stein et al. 2017). Field-collected samples of different populations across Europe (Stein et al. 2017) and within Germany (Kazemi-Dinan et al. 2015b) revealed a particularly high within-species variation in foliar concentrations such as Cd, Zn and Pb. Furthermore, within the latter field survey, populations showed different glucosinolate profiles, forming two distinct chemotypes. In plants of one chemotype, in which 8-methylsulfinyloctyl glucosinolate (‘8MSOO chemotype’) is prominent, a negative correlation was found between foliar Zn and total glucosinolate concentrations (Kazemi-Dinan et al. 2015b). However, the extent to which *A. halleri* also accumulates Si, and how this may impact on leaf concentrations of other elements and glucosinolates, has not been studied until now.

The present study aimed to investigate the effects of Cd, Zn and Si supply on foliar defence traits in *A. halleri*. Individuals of *A. halleri* were collected from three sites, one non-/low-contaminated and two moderately to highly Cd-, Zn- and Pb-contaminated sites in Germany (hereafter ‘accessions’) (Kazemi-Dinan et al. 2015b), maintained under controlled greenhouse conditions for a few years, and subsequently used in the present study. Several cuttings were prepared per accession and grown in a full-factorial design either on unamended or Cd- and Zn-amended soil that was additionally not supplemented or supplemented with Si. After 8 weeks of growth under standardised conditions, we analysed the foliar concentrations of multiple elements and glucosinolates and tested for correlations between Cd, Zn, Si and total glucosinolates. Furthermore, we measured plant phenotypic responses, including foliar trichome density and shoot biomass. We hypothesised that foliar concentrations of Cd and Zn are higher in plants grown on metal-amended than unamended soil without Si supply, but lower in plants supplemented with Si. Such alleviating effects of Si on metal excess have been previously found (Azam et al. 2021; Zheng et al. 2023). We furthermore expected that foliar concentrations of Si are higher in plants supplemented with Si in both unamended and metal-amended plants. Foliar concentrations of other elements, such as Fe, K, Mn and S, which are known to be influenced by metal exposure (Clemens 2001; Andresen et al. 2018), were expected to be affected likewise. Moreover, we expected accession-specific responses in concentrations of elements and glucosinolates. Correlations between Cd, Zn and Si were expected to be positive, but negative correlations were expected between the concentrations of these metal(loid)s and total glucosinolates in line with the trade-off hypothesis (Boyd 2013). The highest densities of foliar trichomes were expected to occur in plants growing on metal-amended soil with Si, because some metal(loid)s are stored and sequestered in trichomes (Zhao et al. 2000; Abe 2019). Finally, shoot biomass was hypothesised to be lower in plants growing on metal-amended than unamended soil, but Si supplementation may mitigate an adverse effect of metal amendment (Azam et al. 2021).

## Materials and methods

### Plant origin, soil treatments, growth conditions and harvest

Plants of *A. halleri* were originally collected from each of three sites in Germany differing in soil pH, soil type and soil concentrations of Cd, Pb and Zn (Kazemi-Dinan et al. 2015b; Stein et al. 2017), namely Wallenfels (Wall: N50°16′1.83″; E11°30′41.26″, non-/low-contaminated site with concentrations of soil Cd: 0.17 and Zn: 5.6 µg/g dry weight on average, collected in 2014–2015), Bestwig (Best: N51°18′27.31″; E8°24′36.17″, moderately contaminated site with concentrations of soil Cd: 2.35 and Zn: 36.16 µg/g dry weight on average, collected in 2017) and Langelsheim (Lan: N51°56′34.22″; E10°20′56.08″, highly contaminated site with concentrations of soil Cd: 0.92 and Zn: 138.56 µg/g dry weight on average, collected in 2020). More detailed soil physicochemical properties of these sites can be found in Kazemi-Dinan et al. (2015b). These plants (hereafter called *accessions*) were maintained and propagated vegetatively under controlled greenhouse conditions at Ruhr University Bochum and were transferred to the greenhouse at Bielefeld University in 2022.

We used a full-factorial design with the factors accession (Wall, Best, Lan), metal amendment (unamended or metal-amended with Cd and Zn) and Si supplementation (−Si and +Si), resulting in four different group treatments per accession. From each plant accession, 40 cuttings were taken (initial *n* = 10 cuttings/group treatment) and half of them transferred individually to pots (70 × 70 × 80 mm) filled with unamended soil, the other half to pots filled with metal-amended soil. To prepare the soil, a 2:1 mixture of P-type soil (lower nitrogen (N)/phosphorus (P)/K content, HAWITA, Germany) and sand was steam-sterilised at 110 °C for 7 h and cooled down overnight. The soil mixture contained low concentrations of bioavailable Si (0.077 ± 0.005 mg/g, mean ± SE; *n* = 9). For metal amendment, 1.1085 mL of CdCl_2_ (resulting in 5 ppm) and 22.5 mL of ZnCl_2_ ( resulting in 300 ppm; Arcos Organics, USA) were added to 1.5 L of the soil, filled up with 700 mL ultrapure water in screw-top PE bottles and mixed overnight in an overhead shaker (Heidolph Instruments GmbH & Co. KG, Schwabach, Germany). Metals were added to the soil as chloride salts and not in another form (e.g. ZnSO_4_) to prevent potential changes in the concentration of S, which could affect the synthesis of glucosinolates in the plant. Subsequently, the soil was dried in an oven at 70 °C for 72 h until most of the water had evaporated, but the final texture of the soil was still relatively moist. After cooling down for another 48 h at a room temperature, this soil mixture was used as growth medium.

Plants were grown in a phytochamber (Percival, Wertingen, Germany) at 22 °C, 60% relative humidity and an 8:16 h light:dark rhythm. The position of the pots was randomised once a week. For the initial 3 weeks, the plants were watered with 5–10 mL demineralised water per pot. From the fourth week onwards, half of the pots per metal treatment were supplemented with a 1.7 mM KCl_2_ (Applichem, Germany) solution (−Si), the other half with a 2 mM K_2_SiO_3_ (Carl Roth, Germany) solution (+Si) twice per week with around 40 mL per pot, similar as in Putra et al. (2021). The pH of the +Si solution was adjusted with HCl (Fisher Scientific, UK) to the pH of the −Si solution (approximately 7).

At the end of an 8 week growth period (thereof 5 weeks without/with Si supplementation), shoots were cut, weighed, and young and old leaves were separated following the rosette architecture of the plant from the inside to the outside, respectively. The leaf material was frozen in liquid N, stored at −80 °C, lyophilised and weighed. For shoot dry biomass, dry biomass of young and old leaves was summed. Due to insufficient sample amount of young leaves, only old leaf samples could be processed for further chemical analyses of element and glucosinolate concentrations (see below). One random old leaf per plant from the outer rosette was sampled and stored at −20 °C to later count the trichomes, as described below. Lyophilised samples were ground in a mill (MM301; Retsch GmbH, Haan, Germany) with 5.0 mm zirconium oxide beads (Cayman Chemical Company, Ann Arbor, USA). In addition, soil samples (*n* = 3 per group treatment) close to the root zones of the plants were collected, oven-dried at 40 °C for 72 h and sieved with a 1.5 mm mesh to determine their element composition and pH.

### Elemental analysis of leaves and soil, and pH determination of the soil

Subsamples of ground leaf and soil material were taken to quantify concentrations of elements. Samples were digested in concentrated HNO_3_ in a microwave (MARSXpress; CEM Microwave Technology Ltd, Matthews, NC, USA) at 190 °C and 1600 W for 20 min, dissolved in ultrapure water and elements measured using inductively coupled plasma atomic emission spectroscopy (ICP-OES) (iCAP 6500 duo, Thermo Fisher, Dreieich, Germany) as described earlier (Stein et al. 2017). For quality controls of the analysis, we included calibration standard solutions for multi-elemental analysis (AnalytiChem GmbH, Duisburg, Germany), 1000 ppm Si as SiO_2_ (Hach Lange GmbH, Düsseldorf, Germany), some certified reference materials for leaf (Polish Virginia tobacco leaves) or soil material (Hard Rock Mine Waste-2780) and *A. halleri* foliar tissues from a non-metalliferous and a metalliferous soil as internal laboratory reference materials (following the detailed method S1 in Stein et al. 2017).

Three grams from each of the sieved samples were processed for determining pH of soil solution with 0.02 M CaCl_2_.2H_2_O (BioChemica, Darmstadt, Germany) at 20 °C using a pH metre (pH 50+ DHS, DOSTMANN electronic GmbH, Germany), following a method described in Kazemi-Dinan et al. (2015b).

### Glucosinolate analysis

Subsamples of the ground leaf material were extracted three times with 80% (v/v) methanol, adding *p*-hydroxybenzyl glucosinolate (Phytoplan Diehm & Neuberger, Germany) as an internal standard at the first extraction. After centrifugation, supernatants were applied on ion-exchange columns of diethylaminoethyl Sephadex A25 (Sigma Aldrich; 0.1 g Sephadex in 2 mL of 0.5 M acetic acid buffer, pH 5) and columns washed. Glucosinolates were incubated overnight with purified *Helix pomatia* sulfatase (Sigma-Aldrich; in 0.02 M acetic acid buffer) and the resulting desulfoglucosinolates were analysed using high-performance liquid chromatography (HPLC) coupled with a diode array detector (Dionex Ultimate 3000, Thermo Fisher Scientific, Waltham, MA, USA) on a Supelcosil LC 18 column (3 μm, 150 × 3 mm, Supelco, USA), as in Barber and Müller (2021). A gradient of eluent A (ultrapure water) and eluent B (methanol) at a flow rate of 0.35 mL/min was used, ramping from 5 to 40% B within 7.0 min, increasing to 60% B until 9 min, to 95% B until 13 min and 95% B were then hold for another 5 min followed by a column cleaning and equilibration cycle. Glucosinolates were identified by comparing their retention times and spectra to an in-house databank. For quantification, peak areas were integrated at 229 nm, related to dry mass and response factors 1, 0.5 and 0.26 for aliphatic, benzenic and indole glucosinolates, respectively, were applied.

### Trichome density

Leaf discs (diameter 4 mm, area 12.6 mm^2^) were taken from the old leaves that had been kept at −20 °C and trichomes on their lower (abaxial) and upper (adaxial) sides counted under a binocular microscope (Olympus SZX16, Japan). Numbers from both leaf sides were summed up per sample.

### Statistical analyses

**Fig. 1:**
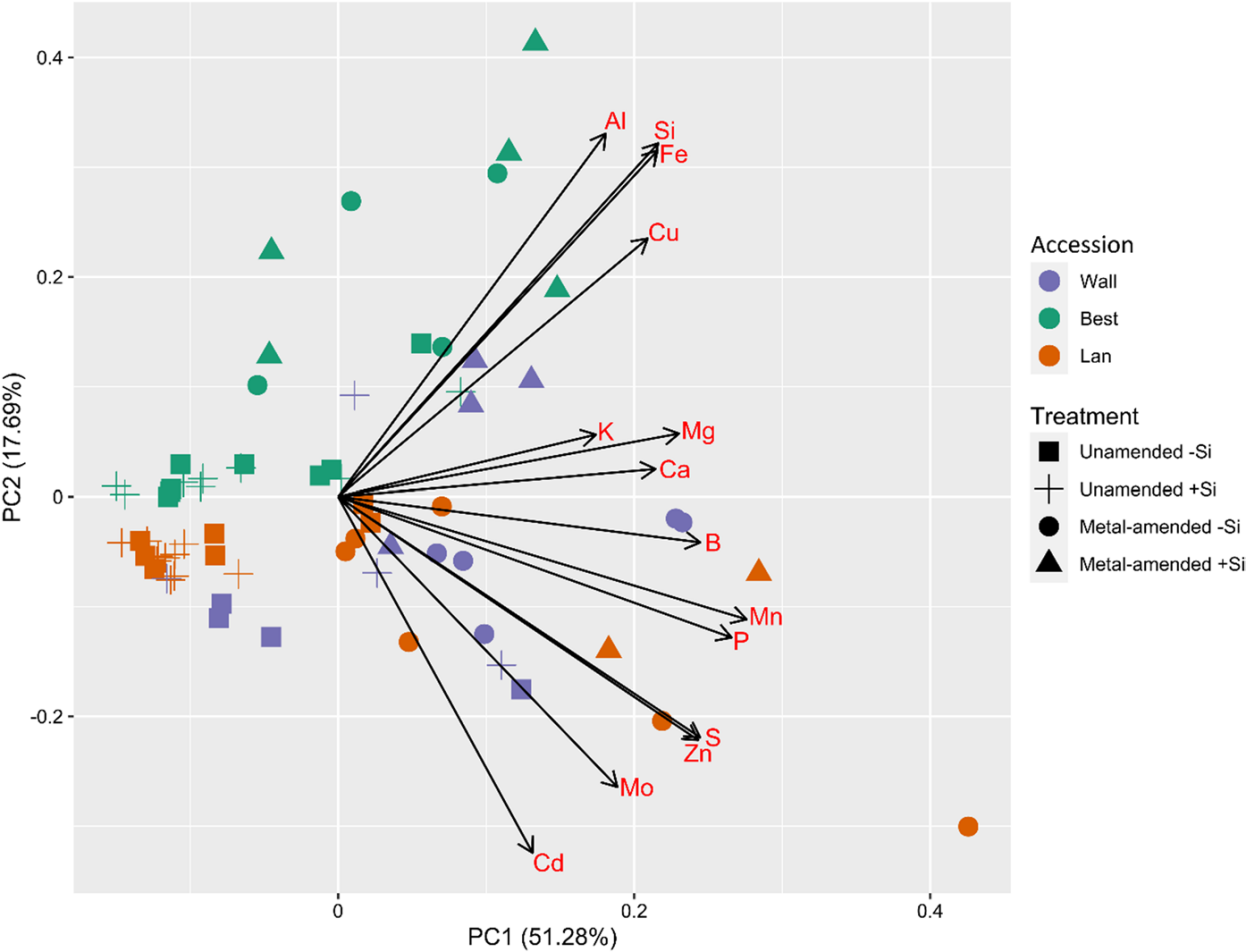
Score plot of principal component analysis (PCA) of 14 elements found in leaves of three accessions of *Arabidopsis halleri* (Wall: Wallenfels, Best: Bestwig and Lan: Langelsheim) grown in soil without or with metal amendment and metalloid (Si) supplementation. Scores (coloured symbols) and loadings (arrows) are presented

All analyses were conducted in R version 4.0.5 (R Core Team 2021). To visualise the global patterns in foliar concentrations of elements and glucosinolates, separately, in the samples from the three accessions grown in the four group treatments, unsupervised principle component analyses (PCA) were performed using ‘prcomp’ (‘ggfortify’ package in Tang et al. 2016; ‘devtools’ package in Wickham et al. 2021). To test whether each of the factors accession, metal amendment and metalloid supplementation and their three-way-interaction contributed significantly (*P* < 0.05) to those patterns, permutational multivariate analyses of variance (PERMANOVA) were performed using ‘adonis’ from the ‘vegan’ package with the Bray–Curtis dissimilarity (Oksanen et al. 2020). PERMANOVA was chosen because the residual models of foliar elements as well as glucosinolates did not fulfil the criterion for normality using a Shapiro–Wilk normality test (‘shapiro.test’). PERMANOVA analyses revealed that accession had a significant effect on those patterns (see Fig. 1. and Fig. 4). Thus, the response variables were subsequently analysed for each accession separately. Generalised linear models (GLM) using ‘glm’ from the ‘stats’ package (R Core Team 2021) with ‘gaussian’ family, followed by ‘Anova’ function (type = ‘III’ to account for unbalanced sample sizes across group treatments) from the ‘car’ package (Fox and Weisberg 2019) with ‘fdr’-adjusted *P*-values were applied to analyse the effects of metal amendment and metalloid supplementation on the concentrations of individual element, individual glucosinolate as well as total glucosinolate concentrations, using total shoot dry biomass as a covariate. Models were also calculated without the covariate and the model with a lower Akaike information criterion (AIC) was chosen for each of the elements and glucosinolates. To test for potential relationships between foliar concentrations of Cd, Zn and Si and total glucosinolate concentrations in the three plant accessions, correlation analyses were calculated using ‘stat_cor’ with the ‘kendall’ method from the ‘ggpubr’ package (Kassambara 2023). Discrete data (count) for foliar trichome density were compared between the group treatments using ‘glm’ and ‘Anova’ (type = ‘III) with ‘poisson’ family. Linear models (LM) using ‘lm’ from the ‘stats’ package (R Core Team 2021), accompanied by ‘Anova’ function (type = ‘III’) from the ‘car’ package (Fox and Weisberg 2019) were computed to test the effects of the factors (metal amendment, metalloid supplementation and their interactions) on shoot dry biomass for each accession. Visual and numerical assessments were done to check for normality (‘qqPlot’ and ‘shapiro.test’) and homogeneity of variance (‘residualPlot’ and ‘leveneTest’) of the residual of LM models. Log_*e*_ data transformation for shoot dry biomass was done in the Wall and Lan accessions, whereas generalised linear model analysis was applied for this trait in the Best accession because the residual of the LM model was neither normally distributed nor homogenous in the latter dataset. When significant (*P* < 0.05) interactions of the main factors on response variables occurred, post-hoc multiple comparison tests using Tukey’s HSD (honestly significant difference) were performed, with ‘pairs’ and ‘cld’ functions from the ‘multcomp’ package (Hothorn et al. 2021). Finally, data visualisation was done using ‘ggplot’ from the ‘ggplot2’ package (Wickham 2016). Raw data together with mean, standard error (SE) and 95% CI (lower limit, upper limit) values of all response variables were provided in Table S2–S8.

## Results

### Effects of experimental factors on foliar elements

In the leaves, 14 elements were detected. When plotted in a PCA, the first two principle components explained 68.97% of the variance. Distinct element compositions were found particularly among the plant accessions and between the metal treatments (Fig. 1). The PERMANOVA revealed a significant three-way interaction among accession, metal treatment and metalloid supplementation (*df* = 2, *F* = 2.54, *P* = 0.04) as well as a significant two-way interaction between accession and metal treatment (*df* = 2, *F* = 3.98, *P* = 0.007).

**Fig. 2:**
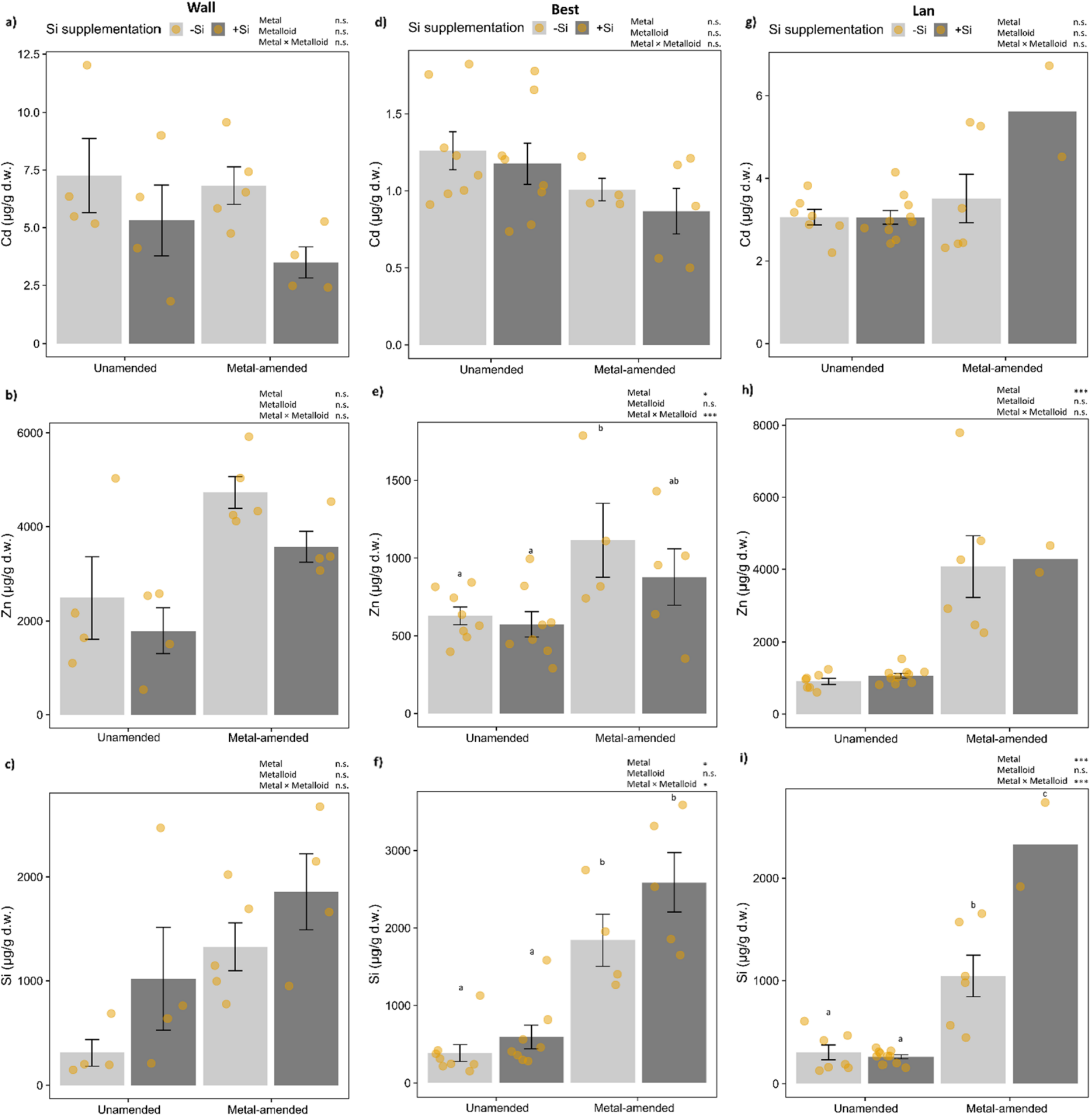
Foliar concentrations (mean ± SE in µg/g d.w.) of Cd, Zn and Si in plants of three accessions of *Arabidopsis halleri* (Wall: Wallenfels, Best: Bestwig and Lan: Langelsheim) grown on soil without or with metal amendment and metalloid (Si) supplementation. Solid circles indicate data points: *n* = 4–10 per treatment combination, except *n* = 2 for the Lan accession with metal amendment and +Si supplementation. Statistical outcomes are indicated as: ∗∗∗*P* < 0.001, ∗*P* < 0.05 and n.s. *P* > 0.1 (non-significant). Different letters above the bars indicate significant differences based on the Tukey’s HSD post-hoc test

In plants of the Wall accession, neither metal nor metalloid treatments resulted in significant changes in foliar concentrations of Cd, Zn and Si (Fig. 2a–c; Table 1). In plants of the Best accession, concentrations of Zn and Si, but not Cd, were significantly influenced by both the metal amendment alone and the interaction of metal amendment and metalloid supplementation (Fig. 2d–f; Table 1). Zn was on average (across all plants) 1.7 times higher in plants with metal amendment (increased by 65.84%) and highest in metal-amended plants −Si. Si was on average 1.5 times higher in Si-supplemented plants grown on unamended soil (increased by 53.69%) and 1.4 times higher in plants grown on metal-amended soil (increased by 40.5%). In plants of the Lan accession, foliar concentrations of Zn were significantly higher, about threefold, in plants grown in metal-amended soil compared to plants grown on unamended soil (Fig. 2h; Table 1). Foliar concentrations of Si were rather low in all plants grown on unamended soil, but significantly higher in plants grown on metal-amended soil −Si and highest in plants on that soil +Si (Fig. 2i; Table 1).

**Table 1:** Effects of metal amendment, metalloid (Si) supplementation and their interactions on foliar concentrations of elements (µg/g d.w.) in the three accessions of *Arabidopsis halleri* (Wall: Wallenfels, Best: Bestwig and Lan: Langelsheim) based on a generalised linear model with a Gaussian distribution

Elements per accession	Factor								
	Metal			Metalloid			Metal × Metalloid		
	*df*	*χ*^*2*^	*P*	*df*	*χ*^*2*^	*P*	*df*	*χ*^*2*^	*P*
Wall									
Cd	1	0.07	0.79	1	1.26	0.26	1	0.34	0.56
Zn	1	0.57	0.45	1	2.21	0.14	1	0.01	0.92
Si	1	1.12	0.29	1	0.55	0.46	1	0.0003	0.99
Al	1	16.01	**< 0.001**	1	0.07	0.8	1	0.001	0.97
Fe	1	16.91	**< 0.001**	1	0.39	0.53	1	0.38	0.54
K	1	12.79	**< 0.001**	1	1.2	0.27	1	8.69	**0.003**
Mn	1	14.19	**< 0.001**	1	0.02	0.89	1	2.75	0.1
S	1	1.05	0.31	1	0.02	0.88	1	0.72	0.4
Best									
Cd	1	0.37	0.54	1	0.15	0.69	1	1.91	0.17
Zn	1	6.29	**0.01**	1	0.07	0.8	1	14.72	**< 0.001**
Si	1	5.96	**0.01**	1	0.46	0.5	1	4.31	**0.04**
Al	1	8.32	**0.004**	1	0.36	0.55	1	0.13	0.72
Fe	1	5.61	**0.02**	1	0.18	0.67	1	1.14	0.29
K	1	2.3	0.13	1	0.22	0.64	1	0.04	0.85
Mn	1	18.6	**< 0.001**	1	0.3	0.58	1	0.10	0.75
S	1	0.13	0.72	1	1.48	0.22	1	1.69	0.19
Lan									
Cd	1	2.97	0.08	1	0.58	0.44	1	3.19	0.07
Zn	1	30.39	**< 0.001**	1	0.09	0.76	1	0.00	0.96
Si	1	20.31	**< 0.001**	1	0.08	0.77	1	22.19	**< 0.001**
Al	1	2.97	0.08	1	0.11	0.74	1	6.58	**0.01**
Fe	1	0.47	0.49	1	0.85	0.36	1	14.44	**< 0.001**
K	1	2.96	0.09	1	1.05	0.31	1	4.27	**0.04**
Mn	1	21.09	**< 0.001**	1	0.1	0.75	1	0.64	0.42
S	1	1.04	0.31	1	0.71	0.4	1	4.05	**0.04**

*n* = 4–10 per treatment combination, except *n* = 2 for the Lan accession with metal amendment and +Si supplementation

Significant *P*-values (*P* < 0.05) are highlighted in bold

**Fig. 3:**
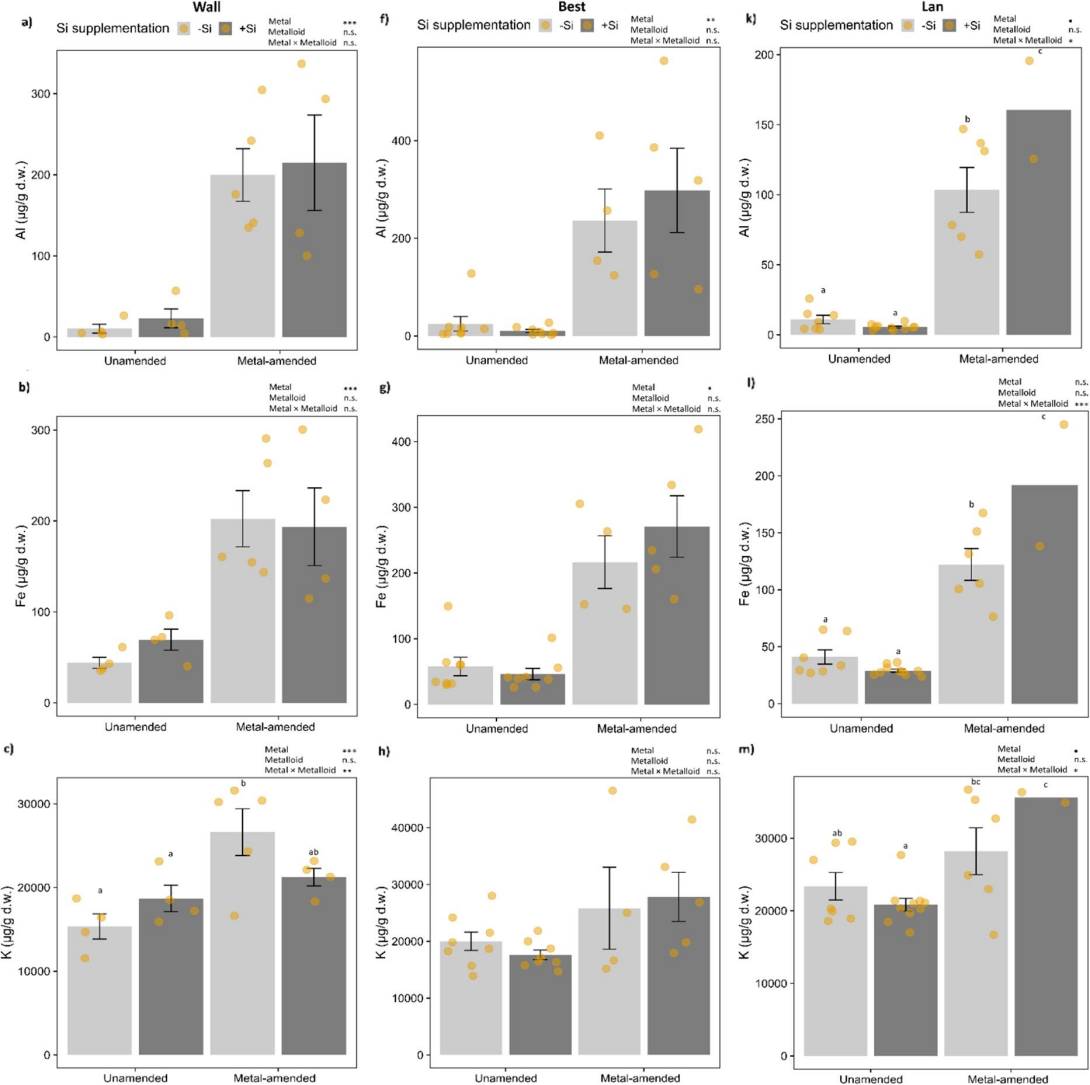

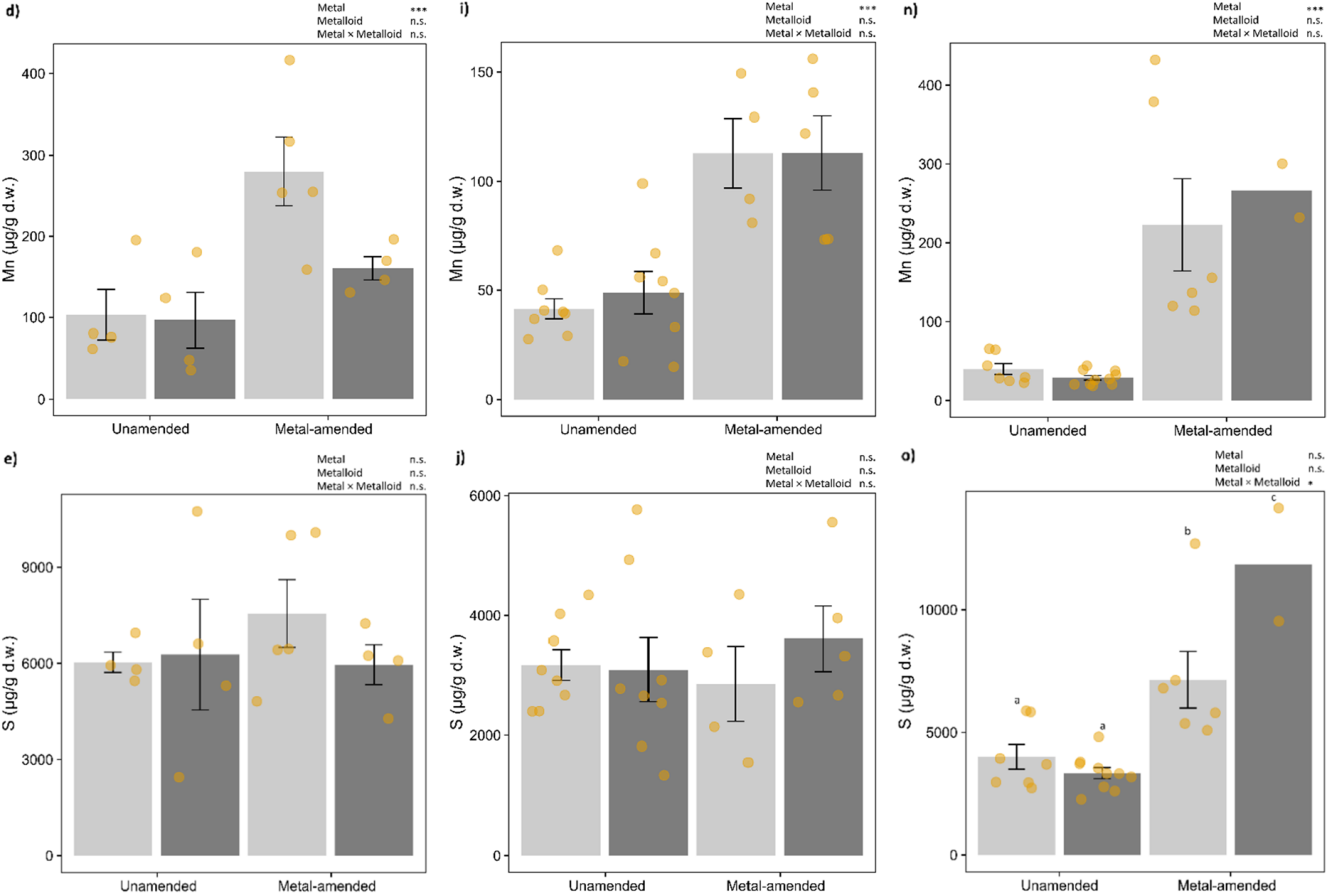
Foliar concentrations (mean ± SE in μg/g d.w.) of Al, Fe, K, Mn and S in plants of the three accessions of *Arabidopsis halleri* (Wall: Wallenfels, Best: Bestwig and Lan: Langelsheim) grown on soil without or with metal amendment and metalloid (Si) supplementation. Solid circles indicate data points:  *n* = 4–10 per treatment combination, except *n* = 2 for the Lan accession with metal amendment and +Si supplementation. Statistical outcomes are indicated as: ∗∗∗*P* < 0.001, ∗∗*P* < 0.01, ∗*P* < 0.05, **•***P *< 0.1 (marginally significant) and n.s. *P* > 0.1 (non-significant). Different letters above the bars indicate significant differences based on the Tukey’s HSD post-hoc test

Besides foliar Cd, Zn and Si, Al, Fe, K, Mn and S were analysed in more detail. In the Wall accession, Al, Fe, K and Mn were significantly higher in the plants growing on metal-amended soil (Fig. 3a–d; Table 1). K concentrations were significantly influenced by an interaction of metal amendment and Si supplementation, whereby plants grown on metal-amended soil −Si had the highest and those grown on unamended soil had the lowest K concentration. In the Best accession, foliar concentrations of Al, Fe and Mn were significantly higher in plants grown on metal-amended soil, but not affected by Si supplementation (Fig. 3f, g, i; Table 1). In the Lan accession, a significant interaction between metal and metalloid treatments was found for foliar concentrations of Al, Fe, K and S, with generally higher levels in plants grown on metal-amended soil and an increase due to +Si supplementation only in those plants (Fig. 3k–m, o; Table 1). For the other elements, see Table S1.

At the end of the experiment, concentrations (mean ± SE) of Cd, Zn and Si in unamended soil were 0.028 ± 0.005, 6.986 ± 1.324 and 59.893 ± 4.231, respectively, whereas concentrations (mean ± SE) of them in metal-amended soil were 0.037 ± 0.003, 10.658 ± 1.036 and 92.857 ± 8.226. Additionally, the pH (mean ± SE) was 6.3 ± 0.05 and 6.5 ± 0.07 in unamended and metal-amended soils, respectively.

### Effects of experimental factors on foliar glucosinolates

**Fig. 4:**
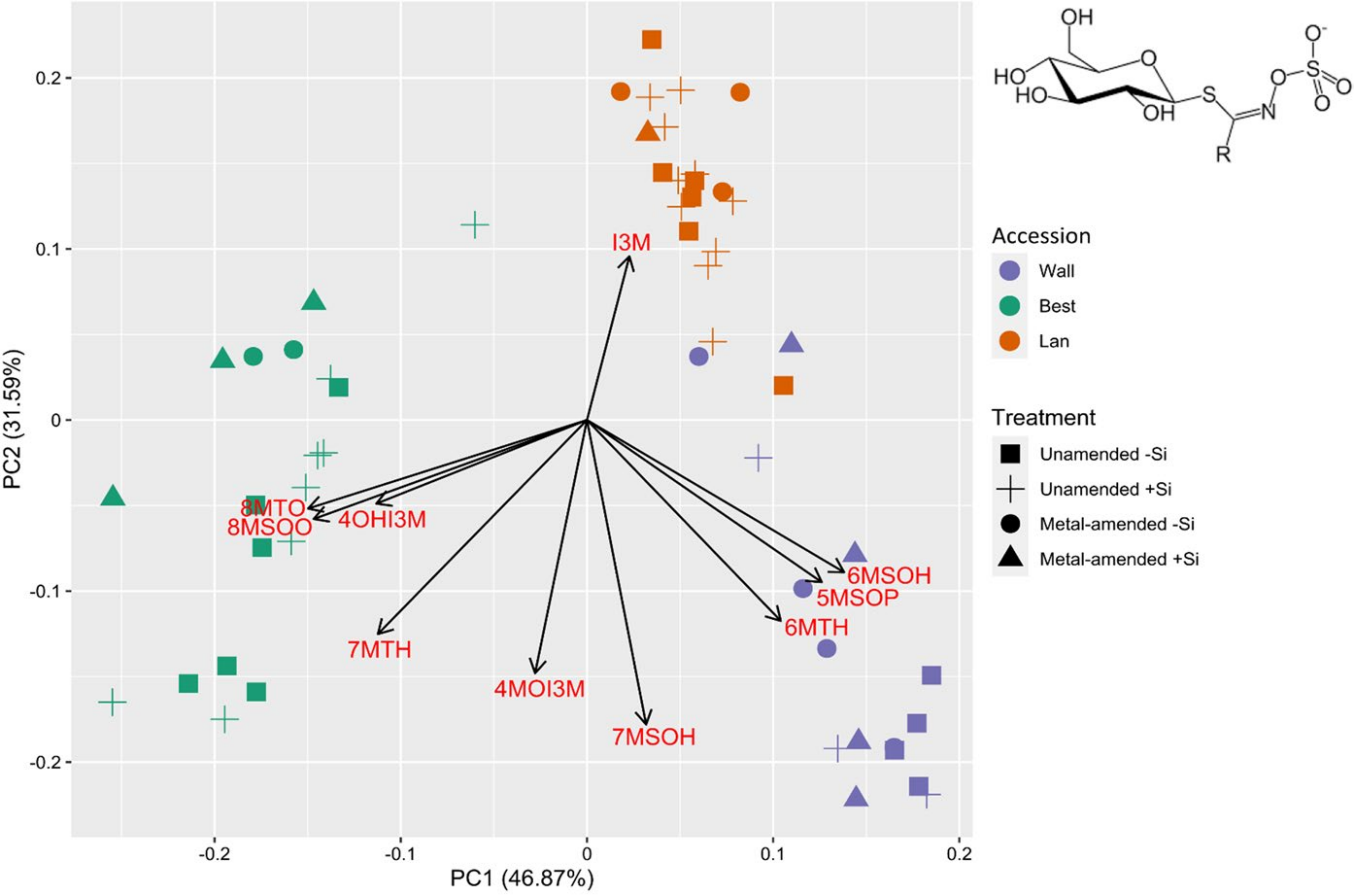
Score plot of principal component analysis (PCA) of 10 glucosinolates found in leaves of three accessions of *Arabidopsis halleri* (Wall: Wallenfels, Best: Bestwig and Lan: Langelsheim) grown on soil without and with metal amendment and metalloid (Si) supplementation. Scores (coloured symbols) and loadings (arrows) are presented. Glucosinolates are abbreviated as: 5MSOP: 5-methylsulfinylpentyl glucosinolate; 6MSOH: 6-methylsulfinylhexyl glucosinolate; 7MSOH: 7-methylsulfinylheptyl glucosinolate; 8MSOO: 8-methylsulfinyloctyl glucosinolate; 6MTH: 6-methylthiohexyl glucosinolate; 7MTH: 7-methylthioheptyl glucosinolate; 8MTO: 8-methylthiooctyl glucosinolate; I3M: indol-3-ylmethyl glucosinolate; 4MOI3M: 4-methoxyindol-3-ylmethyl glucosinolate and 4OHI3M: 4-hydroxyindol-3-ylmethyl glucosinolate. The core structure of glucosinolates is shown in the upper right

**Fig. 5:**
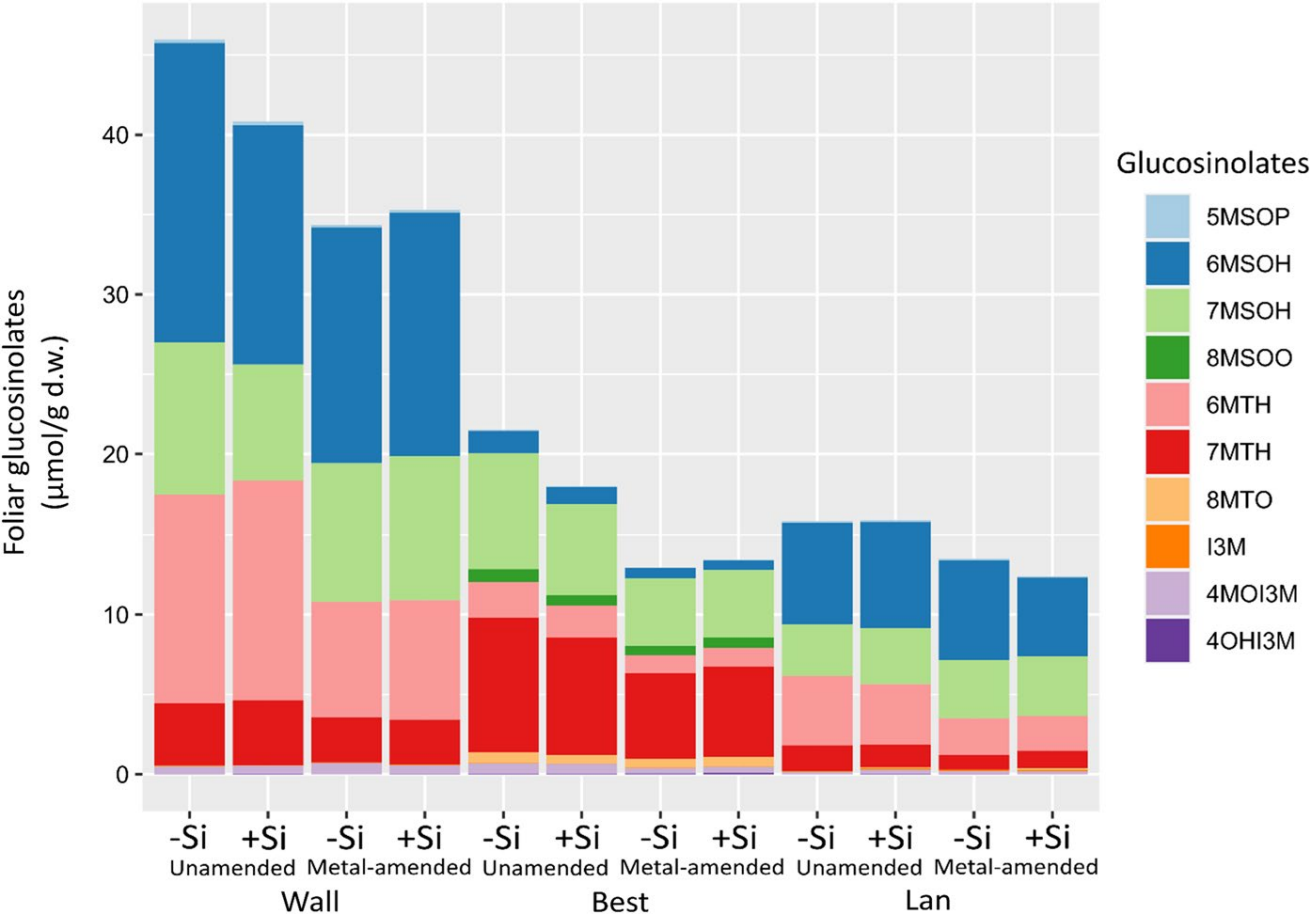
Composition of glucosinolates according to mean concentrations (µmol/g d.w.) found in leaves of three accessions of *Arabidopsis halleri* (Wall: Wallenfels, Best: Bestwig and Lan: Langelsheim) grown on soil without and with metal amendment and metalloid (Si) supplementation. *n* = 3–10 per treatment combination, except *n* = 2 for the Best accession with metal amendment and −Si supplementation and *n* = 1 for the Lan accession with metal amendment and +Si supplementation. Glucosinolates are abbreviated as: 5MSOP: 5-methylsulfinylpentyl glucosinolate; 6MSOH: 6-methylsulfinylhexyl glucosinolate; 7MSOH: 7-methylsulfinylheptyl glucosinolate; 8MSOO: 8-methylsulfinyloctyl glucosinolate; 6MTH: 6-methylthiohexyl glucosinolate; 7MTH: 7-methylthioheptyl glucosinolate; 8MTO: 8-methylthiooctyl glucosinolate; I3M: indol-3-ylmethyl glucosinolate; 4MOI3M: 4-methoxyindol-3-ylmethyl glucosinolate and 4OHI3M: 4-hydroxyindol-3-ylmethyl glucosinolate

In total, ten glucosinolates could be detected in the leaves, of which seven were aliphatic and three indole glucosinolates. The first two components of the PCA explained 78.46% variance of the global foliar glucosinolate composition and revealed a clear separation among the accessions, as also confirmed by the PERMANOVA (*df* = 2, *F* = 78.69, *P* = 0.001). Plants of the Best accession showed the most distinct pattern (Fig. 4). 5-Methylsulfinylpentyl glucosinolate (5MSOP), 6-methylsulfinylhexyl glucosinolate (6MSOH) and 6-methylthiohexyl glucosinolate (6MTH) were particularly high in plants of the Wall accession; 8-methylsulfinyloctyl glucosinolate (8MSOO), 8-methylthiooctyl glucosinolate (8MTO) and 4-hydroxyindol-3-ylmethyl glucosinolate (4OHI3M) in those of the Best and indol-3-ylmethyl glucosinolate (I3M) in those of the Lan accession (Figs. 4 and 5). In fact, 8MSOO could be exclusively found in plants of the Best accession (Fig. 5). The PERMANOVA also revealed a significant effect of metal amendment on the global pattern of foliar glucosinolates (*df* = 1, *F* = 4.06, *P* = 0.018). The total concentration of all glucosinolates was highest in plants of the Wall accession (Fig. 5).

In the Wall accession, Si supplementation led to a significant increase in foliar concentrations of 4OHI3M in plants grown on unamended soil (Fig. S1a; Table 2), but no effect was found for total glucosinolates (Fig. S1b; Table 2). In the Best accession, metal amendment led to significant decreases in foliar concentrations of 5MSOP, 6MSOH and 7-methylsulfinylheptyl glucosinolate (7MSOH) by almost two-fold, particularly for 5MSOP and 6MSOH (Fig. S1c−e; Table 2). Si supplementation had marginally significant effects on the foliar concentrations of 6MSOH and 7MSOH, with reduced concentrations found in +Si plants. Foliar concentrations of total glucosinolates were significantly lower in the plants grown on metal-amended soil (Fig. S1f; Table 2). In the Lan accession, all three detected indole glucosinolates were significantly higher in plants growing in Si-supplemented soil (Fig. S1h–j; Table 2), while low concentrations of 8MTO were detected in plants supplemented with Si of this accession (Fig. S1g; Table 2), but no effect was found for total glucosinolates (Fig. S1k; Table 2).

**Table 2:** Effects of metal amendment, metalloid (Si) supplementation and their interactions on foliar concentrations of glucosinolates (µmol/g d.w.) in the three accessions of *Arabidopsis halleri* (Wall: Wallenfels, Best: Bestwig and Lan: Langelsheim) based on a generalised linear model with a Gaussian distribution

Glucosinolates per accession	Factor								
	Metal			Metalloid			Metal × Metalloid		
	*df*	*χ*^*2*^	*P*	*df*	*χ*^*2*^	*P*	*df*	*χ*^*2*^	*P*
Wall									
5MSOP	1	2.87	0.09	1	2.05	0.15	1	1.91	0.17
6MSOH	1	2.67	0.10	1	1.59	0.21	1	3.24	0.07
7MSOH	1	0.51	0.48	1	3.18	0.07	1	2.28	0.13
6MTH	1	3.05	0.08	1	0.04	0.85	1	0.01	0.93
7MTH	1	1.03	0.31	1	0.03	0.87	1	0.02	0.90
I3M	1	2.74	0.10	1	0.08	0.78	1	0.27	0.60
4MOI3M	1	0.88	0.35	1	0.01	0.92	1	0.17	0.68
4OHI3M	1	0.31	0.58	1	8.00	**0.005**	1	2.05	0.15
Total glucosinolates	1	2.58	0.11	1	0.43	0.51	1	0.33	0.57
Best									
5MSOP	1	6.80	**0.009**	1	1.14	0.29	1	0.26	0.61
6MSOH	1	7.07	**0.008**	1	3.39	0.066	1	0.69	0.41
7MSOH	1	6.55	**0.01**	1	3.63	0.057	1	0.95	0.33
8MSOO	1	1.71	0.19	1	2.60	0.11	1	1.36	0.24
6MTH	1	2.96	0.09	1	0.29	0.59	1	0.11	0.74
7MTH	1	2.47	0.12	1	0.7	0.4	1	0.29	0.59
8MTO	1	0.83	0.36	1	2.97	0.08	1	1.18	0.28
I3M	1	1.34	0.25	1	0.29	0.59	1	0.35	0.55
4MOI3M	1	3.59	0.06	1	0.02	0.88	1	0.11	0.74
4OHI3M	1	0.47	0.49	1	0.02	0.89	1	0.56	0.45
Total glucosinolates	1	4.21	**0.04**	1	1.57	0.21	1	0.53	0.47
Lan									
5MSOP	1	0.03	0.85	1	1.23	0.27	1	1.79	0.18
6MSOH	1	0.01	0.93	1	0.07	0.78	1	0.41	0.52
7MSOH	1	0.48	0.49	1	0.37	0.54	1	0.04	0.84
6MTH	1	2.38	0.12	1	0.32	0.57	1	0.04	0.85
7MTH	1	2.08	0.15	1	0.33	0.57	1	0.19	0.66
8MTO	1	0	1	1	8.55	**0.003**	1	1.02	0.31
I3M	1	7.17	**0.007**	1	9.86	**0.002**	1	3.51	0.06
4MOI3M	1	0.45	0.5	1	21.06	**< 0.001**	1	3.24	0.07
4OHI3M	1	0.56	0.45	1	6.00	**0.01**	1	3.76	0.053
Total glucosinolates	1	0.42	0.52	1	0.0005	0.98	1	0.03	0.86

*n* = 3–10 per treatment combination, except *n* = 2 for the Best accession with metal amendment and −Si supplementation and *n* = 1 for the Lan accession with metal amendment and +Si supplementation

Significant *P*-values (*P* < 0.05) are highlighted in bold

Glucosinolates are abbreviated as:* 5MSOP* 5-methylsulfinylpentyl glucosinolate,* 6MSOH* 6-methylsulfinylhexyl glucosinolate,* 7MSOH* 7-methylsulfinylheptyl glucosinolate,* 8MSOO* 8-methylsulfinyloctyl glucosinolate,* 6MTH* 6-methylthiohexyl glucosinolate,* 7MTH* 7-methylthioheptyl glucosinolate,* 8MTO* 8-methylthiooctyl glucosinolate,* I3M* indol-3-ylmethyl glucosinolate,* 4MOI3M* 4-methoxyindol-3-ylmethyl glucosinolate,* 4OHI3M* 4-hydroxyindol-3-ylmethyl glucosinolate

### Correlation between foliar metal(loid)s and glucosinolates

Positive correlations were found between foliar concentrations of Zn and Si in the three plant accessions (Fig. S2a) and between Zn and Cd in the Best and Lan accessions (Fig. S2c). However, no correlation was found between Cd and Si in the three accessions (Fig. S2b). A positive correlation was found between foliar concentrations of Cd and total glucosinolates only in the Best accession (Fig. S2e), whereas no correlation was observed between Zn or Si and total glucosinolates in the three accessions (Figs. S2d and S2f).

### Effects of experimental factors on foliar trichome density and shoot biomass

**Fig. 6:**
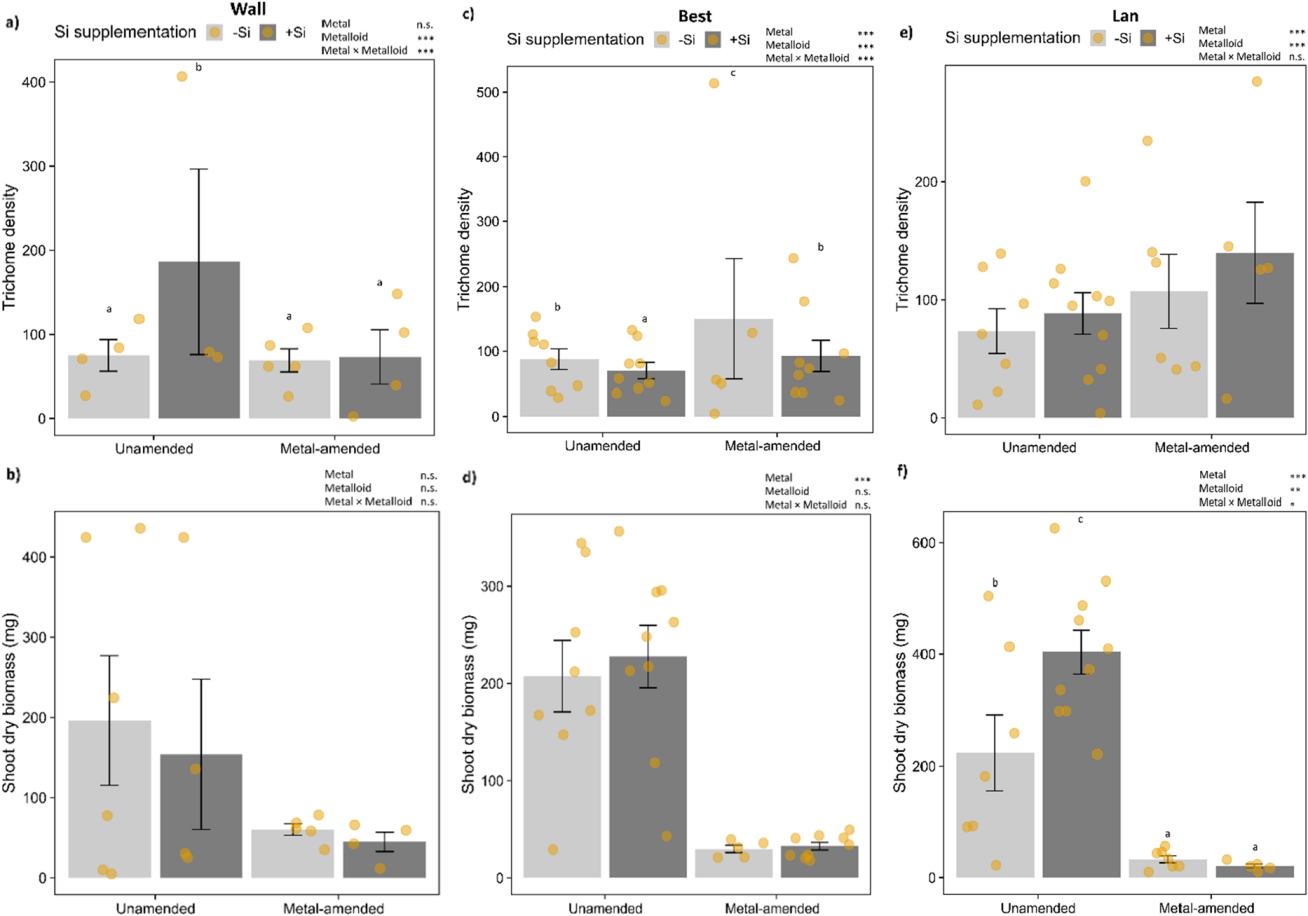
Foliar trichome density (mean ± SE of count per 12.6 mm^2^) and shoot dry biomass (mean ± SE in mg) in plants of the three accessions of *Arabidopsis halleri* (Wall: Wallenfels, Best: Bestwig and Lan: Langelsheim) grown on soil without and with metal amendment and metalloid (Si) supplementation. Solid circles indicate data points: *n* = 3–10 per treatment combination. Statistical outcomes are indicated as: ∗∗∗*P* < 0.001, ∗∗*P* < 0.01, ∗*P* < 0.05 and n.s. *P* > 0.1 (non-significant). Different letters above the bars indicate significant differences based on the Tukey’s HSD post-hoc test

The foliar trichome density showed quite distinct patterns across the three accessions. In plants of the Wall accession, the trichome density was 2.6 times higher in plants grown on unamended soil +Si compared to the plants of the other three treatments (Fig. 6a; Table 3). In the Best accession, the trichome density was significantly influenced by metal amendment, Si supplementation and their interaction, with somewhat higher numbers of trichomes per area in −Si plants, particularly in plants grown on metal-amended soil (Fig. 6c; Table 3). In plants of the Lan accession, the trichome density was significantly higher in plants grown on metal-amended than unamended soil and in plants that experienced Si supplementation compared to −Si plants (Fig. 6e; Table 3).

**Table 3:** Effects of metal amendment, metalloid (Si) supplementation and their interactions on total trichome density (count per 12.6 mm^2^) and shoot dry biomass (mg) in the three accessions of *Arabidopsis halleri* (Wall: Wallenfels, Best: Bestwig and Lan: Langelsheim) based on a generalised linear model with a Poisson distribution and a linear model for the Wall and Lan accessions and a generalised linear model with a Gaussian distribution for the Best accession, respectively

Phenotypic responses per accession	Factor								
	Metal			Metalloid			Metal × Metalloid		
Wall	*df*	*χ*^*2*^	*P*	*df*	*χ*^*2*^	*P*	*df*	*χ*^*2*^	*P*
Total trichome density	1	1.11	0.29	1	171.54	**< 0.001**	1	64.27	**< 0.001**
	*df*	*F*	*P*	*df*	*F*	*P*	*df*	*F*	*P*
(log_*e*_) Shoot dry biomass	1	0.08	0.77	1	0.02	0.90	1	0.20	0.66
Best	*df*	*χ*^*2*^	*P*	*df*	*χ*^*2*^	*P*	*df*	*χ*^*2*^	*P*
Total trichome density	1	105.28	**< 0.001**	1	16.54	**< 0.001**	1	12.29	**< 0.001**
	*df*	*χ*^*2*^	*P*	*df*	*χ*^*2*^	*P*	*df*	*χ*^*2*^	*P*
Shoot dry biomass	1	17.46	**< 0.001**	1	0.31	0.58	1	0.10	0.75
Lan	*df*	*χ*^*2*^	*p*	*df*	*χ*^*2*^	*p*	*df*	*χ*^*2*^	*p*
Total trichome density	1	41.17	**< 0.001**	1	11.36	**< 0.001**	1	1.06	0.3
	*df*	*F*	*P*	*df*	*F*	*P*	*df*	*F*	*P*
(log_*e*_) Shoot dry biomass	1	22.35	**< 0.001**	1	8.46	**0.008**	1	6.97	**0.01**

*n* = 3–10 per treatment combination

Shoot dry biomass was significantly lower in plants of the Best and Lan accessions when plants were grown on metal-amended compared to those grown on unamended soil (Fig. 5d and f; Table 3). In the Lan accession, shoot biomass was also significantly affected by Si and the interaction of metal amendment and Si supplementation, with Si supplementation (+Si) leading to an increase in shoot biomass notably in plants grown on unamended soil by almost two-fold (Fig. 6f).

## Discussion

In this study, we showed that soil metal amendment with Cd and Zn and Si supplementation influenced various potential foliar defence traits, such as elemental (Zn and Si), organic (glucosinolates) and mechanical (trichomes) defences in the hyperaccumulator *A. halleri* under controlled growth conditions. Moreover, there was a high within-species variation in such defence responses among the three accessions originating from non-/low- (Wall), moderate- (Best) and high-metalliferous (Lan) sites within Germany. These accessions also exhibited distinct glucosinolate profiles, indicating chemotypes. Particularly, 8MSOO was specific for the Best accession, corroborating the chemotype found in a previous field study (Kazemi-Dinan et al. 2015b). Foliar concentrations of certain glucosinolates and their sum were significantly correlated with foliar concentrations of Cd, Zn or Si, depending on accession origin.

We had expected that *A. halleri* plants show increased foliar concentrations of Cd, Zn and Si when grown on soil amended or supplemented with those metal(loid)s in comparison to control plants. Surprisingly, Cd concentrations were not enhanced in the leaves and the level remained below the threshold for Cd hyperaccumulation, which is 100 µg/g d.w. (Krämer 2010). While several previous studies with *A. halleri* grown under controlled growth chamber conditions on metal-amended soil reported foliar Cd levels above this threshold (Stein et al. 2017; Stolpe et al. 2017; Tewes et al. 2018), foliar concentrations in two accessions (from Hamburg and Langelsheim) had higher Cd levels than plants grown on unamended soil but not above the hyperaccumulation threshold (Kazemi-Dinan et al. 2014). One explanation may be related to different physicochemical properties of the growth substrate used in those experiments. The soil used in our experiment likely contained more organic matter, while in previous experiments low organic loamy soil had been used (Stein et al. 2017; Stolpe et al. 2017; Tewes et al. 2018). High soil organic matter is negatively associated with Cd bioavailability and Cd uptake by plants, possibly through complexation and sorption (Rieuwerts et al. 1998; Violante et al. 2010). Moreover, at the end of experiment, concentrations of soil Cd and Zn were relatively higher on metal-amended than unamended soil, but these seem to be far below concentrations of Cd and Zn on metalliferous sites either in Best or Lan (Kazemi-Dinan et al. 2015b). Another explanation could be that influential pathways like Fe acquisition, which contribute to Cd accumulation, were suppressed because of the elevated leaf Fe levels in these plants cultivated on metal-amended soil (Fig. 3b, g, l). Finally, in our study, old leaves were analysed which usually contain lower concentrations of metals than the younger leaves (Stolpe et al. 2017). Interestingly, plants originating from the Wall accession, and thus the non-/low-metalliferous site, contained the highest concentrations of foliar Cd and Zn relative to the other two accessions in the present study, corroborating previous studies (Bert et al. 2002; Stein et al. 2017).

We did not find significant effects of Si supplementation on the foliar concentrations of Cd and Zn, at least in the old leaves studied here, although Si concentrations increased by 7.5% in +Si-supplemented soil relative to −Si at the end of the experiment. It is still poorly understood how and to what extent Si influences the uptake and accumulation of metals, including Cd and Zn, in metal(loid) hyperaccumulator plants (Putra and Müller 2023). However, in the As hyperaccumulator *I. cappadocica*, supplementation with Si led to a significant reduction of shoot and root concentrations of As and Cd (Azam et al. 2021). In addition, Si has been reported to ameliorate metal and metalloid toxicity, for example in *Arabidopsis* sp. (Khan et al. 2021; Meena et al. 2021). In field-collected foliar samples of *A. halleri* from Langelsheim (Lan), Si was detected together with Zn at the subcellular level, such as in the cell wall, cytoplasm, intercellular space and vacuole in the form of Zn-silicate precipitate or SiO_2_ deposit (Neumann and Zur Nieden 2001; Neumann and De Figueiredo 2002). Whether accumulation or deposition of Si has a functional role in metal tolerance or detoxification in *A. halleri* should be further investigated. The positive correlations between foliar concentrations of Zn and Si found in our experimental plants for the three accessions of *A. halleri* may point to a functional role. The formation of a Si-pectin matrix (Si–O–C linkage) was found to be related to a reduction of La stress in the Al-, La- and Si-accumulating fern *Dicranopteris linearis* (Zheng et al. 2023). In *O. sativa*, concentrations of Si were highest when plants were subjected to Cd and Cu with Si supplementation (Kim et al. 2014). In *A. thaliana* and other Brassicaceous crop species, foliar Si concentrations range from 700 up to 2500 µg/g d.w. when growing on soil supplemented with 1.7 mM K_2_SiO_3_ (Deshmukh et al. 2020). The Si concentrations detected in our experimental plants of *A. halleri* fall into this range.

The foliar concentrations of several other elements also changed in response to either metal amendment alone or the interaction of metal amendment and Si supplementation, with distinct effects among accessions. Plants grown on metal-amended soil showed higher foliar concentrations of Al, Fe and Mn in all three accessions. Al is the third most ubiquitous element in the soil, commonly found in the form of aluminium silicate (Al_2_SiO_5_) (Ofoe et al. 2023). Soil pH could have affected the availability of several elements. For example a soil pH below 5 is known to increase bioavailability of elements, such as Al (Ofoe et al. 2023), Mn and Fe (Violante et al. 2010). The Al concentrations found in our experimental plants are assumed to be in a normal range for most plants and unlikely to cause phytotoxicity (Schmitt et al. 2016). At least in plants of the Lan accession grown on metal-amended soil with Si supplementation, Al concentrations were higher than in plants of the other treatment groups. High accumulation of Al was found to be positively correlated with Si accumulation in the fern *D. linearis* (Liu et al. 2021). Increased concentrations of foliar Fe and Mn might be related to the production of metalloenzymes, such as Fe- and Mn-dependent superoxide dismutases, which act as antioxidant defences to protect cells from damage caused by reactive oxygen species in response to metal stress (Alscher et al. 2002). Other elements, such as K and molybdenum (Mo), are macro- and micronutrients needed for plant growth and are also involved in enzyme activation and redox reactions (Kaiser et al. 2005; Wang et al. 2013). In the Lan accession, increased foliar concentrations of S in plants grown on metal-amended soil were in line with a previous finding (Stolpe et al. 2017). An up-regulation of genes for S metabolism has been found in *N. caerulescens* plants grown hydroponically in response to Cd and Zn excess (Van De Mortel et al. 2008). Increased foliar concentrations of S might be related to metal-induced phytochelatin and glutathione production, which is derived from cysteine and involved as metal-binding protein and antioxidant, respectively (Grill et al. 1989; Freeman et al. 2004).

The pH of our experimental metal-amended soil (average 6.5) was 0.2 higher than that of unamended soil (6.3). Furthermore, the metal-amended soil had higher concentrations of soil Fe, K and Mn at the end of the final harvest of the plants (see Table S4). A slightly higher soil pH and increased levels of these three elements in the metal-amended soil could be related to initial waterlogging (Lu et al. 2004), associated with the metal amendment procedure applied in this experiment. In the three locations where the plants were originally collected, soil pH, soil type and soil concentrations of Cd, Pb and Zn varied strongly (Kazemi-Dinan et al. 2015b). For example, soil pH in Wall was quite acidic (average 4.9) with dark soil type containing minerals and large amounts of organic matter, but with relatively low concentrations of Cd, Pb and Zn. In contrast, soil pH in Best and Lan were much higher (average 5.8 and 6.4, respectively) with a soil-stone mixture and higher concentrations of soil Cd, Pb and Zn than in Wall. Such physicochemical properties may have affected the final elemental concentrations in the aboveground plant material.

With regard to glucosinolates, distinct profiles could be detected in plants of the three populations, as have been found previously in leaves of field-collected plants (Kazemi-Dinan et al. 2015b). Brassicaceae species with distinct chemotypes, such as *A. thaliana*, *Barbarea vulgaris*, *Brassica oleracea* and *Bunias orientalis*, have been found to differ in their defence potential or acceptance towards some herbivores and pathogens (Gols et al. 2008; Arany et al. 2008; Christensen et al. 2019; Tewes and Müller 2020). These chemotypes may be genetically fixed, but differences in the concentrations of individual compounds may also result from responses to certain environmental conditions. On average, the total glucosinolate concentrations were lower in the leaves of plants of all three accessions upon cultivation on metal-amended soil. This pattern, however, was only significant in the Best accession, in which three aliphatic glucosinolates, 5MSOP, 6MSOH and 7MSOH, were lower in plants on such soil. Lower foliar glucosinolate concentrations in plants grown on metal-amended soil may be associated with the intertwined S metabolism, which is needed for glucosinolate biosynthesis and, at least in some species, for metal chelation (Rausch and Wachter 2005; Ernst et al. 2008; Pongrac et al. 2010). Strikingly, foliar concentrations of the indole glucosinolates I3M in Wall and Lan and 4MOI3M and 4OHI3M in plants of the Lan accession were significantly higher in plants with Si supplementation than in those without. Induction of indole glucosinolates in leaves of Brassicaceous plants is readily caused by insect herbivory (Textor and Gershenzon 2009) and breakdown products of indole glucosinolates are known for their various biological effects (Agerbirk et al. 2009). The changes in the glucosinolate profiles in response to metal amendment and Si supplementation may thus potentially affect the feeding behaviour and growth performance of herbivorous insects as well as fungi. Si accumulation also induces foliar concentrations of endogenous jasmonic acid (Hall et al. 2019), which could affect the biosynthesis of glucosinolates (Hopkins et al. 2009). However, whether the foliar concentrations of jasmonic acid are actually altered by metal(loid) treatment in *A. halleri* requires further analysis.

Our correlation analysis indicated no apparent negative correlation between foliar concentrations of Cd, Zn or Si and total glucosinolates in the three plant accessions. These findings thus might not support the trade-off hypothesis, at least in part, because plants grown on metal-amended soil had significantly lower total glucosinolates in the Best accession. The trade-off hypothesis postulates that hyperaccumulators may favour elemental over organic defences, because the synthesis of organic defences may be metabolically costly (Boyd 2007). However, whilst providing a *raison d’être*, this hypothesis should be challenged. First, the analysis of costs and benefits between the two types of defences is difficult and should involve physiological, ecological and evolutionary assessments (Boyd 2013). Second, just like other plants, hyperaccumulators synthesise a plethora of specialised metabolites, and their composition and concentration may differ within species and even within an individual, termed chemodiversity (Moore et al. 2014; Müller and Junker 2022), which is still overlooked in many hyperaccumulators (Putra and Müller 2023). Third, trade-offs may be more relevant in the context of induced resistance (Fones and Preston 2013; Tewes et al. 2018; Fones et al. 2019). Finally, stemming from a trade-off, it cannot be explicitly determined, which one of the two factors is causing a change in the other. Using one of the RNAi line mutants of *A. halleri* incapable of hyperaccumulating Cd and Zn may potentially disentangle this causal vs. consequential relationship.

As another layer of defence, several plants also produce trichomes, which can be highly plastic in response to (a)biotic factors (Dalin et al. 2008), such as insect herbivory, ultraviolet-B radiation as well as metal(loid) exposure (see review in Karabourniotis et al. 2020). For example, ultraviolet-B radiation was found to increase trichome density in *A. thaliana* (Yan et al. 2012). In some hyperaccumulator plants, trichomes are suggested to contribute to metal detoxification and metal sequestration in hyperaccumulators, such as Cd and Zn in *A. halleri* (Zhao et al. 2000; Fukuda et al. 2008), Ni and Mn in some Ni-accumulating *Alyssum* species (Krämer et al. 1997; Broadhurst et al. 2004) and thallium (Tl) in the Tl hyperaccumulator *Biscutella laevigata* (Wierzbicka et al. 2016). In the present experiment, trichome density differed in response to the metal(loid) treatments and the pattern was accession-specific, with a higher density on leaves of plants of the Best and Lan accessions grown on metal-amended soil. Moreover, Si supplementation affected trichome densities in all three accessions, but in different directions, resulting in a reduced trichome density in plants of Best. Silicification often occurs in foliar epidermal cells and many Si-accumulating species deposit a substantial amount of Si in the trichome cells (Kumar et al. 2017; Abe 2019; Nakamura et al. 2022). A lower trichome density could also be associated with a higher diversity of glucosinolates or other specialised metabolites in the Best accession.

Our study suggests that growth on a metal-amended soil might incur a cost in terms of shoot biomass reduction. However, it should be noted that the maximal Zn and Cd concentrations reached in leaves of plants from all of these accessions were far below the toxicity thresholds in *A. halleri* (Becher et al. 2004). It is therefore likely that the reduced biomass observed in *A. halleri* individuals grown on metal-amended soil is unrelated to the Zn and Cd levels in the metal-amended soil used here. Instead, this biomass reduction might coincide with different soil structure of the experimental soils due to their different treatments. In another accession and using slightly different soil conditions, no significant differences were found in shoot dry biomass of *A. halleri* plants grown on unamended versus metal-amended soil for two months under standardised greenhouse conditions (Kazemi-Dinan et al. 2015a). Shoot dry biomass was even significantly higher in plants growing on metal-amended than unamended soil after three months of growth outside (Kazemi-Dinan et al. 2015a). The effect of Si supplementation on shoot biomass was minor in our study; only in the Lan plants grown on unamended soil, Si supplementation led to a higher biomass. In contrast, Si supplementation resulted in an increased shoot biomass in some Si-accumulating plants belonging to the Poaceae and Fabaceae (Detmann et al. 2012; Xu et al. 2020; Putra et al. 2021). Thus, effects of different metal(loid)s may highly depend on the growing substrate and condition, concentration of added metal(loid)s as well as plant species. Reciprocal transplant experiments, as for example, explained in Harrison and Rajakaruna (2011) in addition to controlled standardised experiments, could provide further information regarding local adaptation, adaptive variation and selection towards plant defence traits.

In summary, our study suggests that effects of metal amendment and metalloid supplementation on foliar elemental, organic and mechanical defences are accession-specific in *A. halleri*. Further studies should address in more detail the role of Si, which may modify elemental and organic defences, but also impact on metal tolerance or detoxification in hyperaccumulators (Neumann and Zur Nieden 2001). Finally, further investigation is necessary to test the roles of various putative defence types against different herbivores and/or pathogens in *A. halleri* to understand the eco-evolutionary relevance of investment in distinct defences and potential trade-offs at the population level.

### Supplementary Information

Below is the link to the electronic supplementary material.

Supplementary material 1 (DOCX 1057.2 kb)

Supplementary material 2 (XLSX 55.2 kb)

## References

[CR1] Abe J (2019). Silicon deposition in leaf trichomes of cucurbitaceae horticultural plants: a short report. Am J Plant Sci.

[CR2] Agerbirk N, De Vos M, Kim JH, Jander G (2009). Indole glucosinolate breakdown and its biological effects. Phytochem Rev.

[CR3] Alscher RG, Erturk N, Heath LS (2002). Role of superoxide dismutases (SODs) in controlling oxidative stress in plants. J Exp Bot.

[CR4] Andama JB, Mujiono K, Hojo Y (2020). Nonglandular silicified trichomes are essential for rice defense against chewing herbivores. Plant Cell Environ.

[CR5] Andresen E, Peiter E, Küpper H (2018). Trace metal metabolism in plants. J Exp Bot.

[CR6] Arany AM, de Jong TJ, Kim HK (2008). Glucosinolates and other metabolites in the leaves of *Arabidopsis thaliana* from natural populations and their effects on a generalist and a specialist herbivore. Chemoecology.

[CR7] Azam SK, Karimi N, Souri Z, Vaculík M (2021). Multiple effects of silicon on alleviation of arsenic and cadmium toxicity in hyperaccumulator *Isatis cappadocica* Desv. Plant Physiol Biochem.

[CR8] Barber A, Müller C (2021). Drought and subsequent soil flooding affect the growth and metabolism of savoy cabbage. Int J Mol Sci.

[CR9] Becher M, Talke IN, Krall L, Krämer U (2004). Cross-species microarray transcript profiling reveals high constitutive expression of metal homeostasis genes in shoots of the zinc hyperaccumulator *Arabidopsis halleri*. Plant J.

[CR10] Bert V, Bonnin I, Saumitou-Laprade P (2002). Do *Arabidopsis halleri* from nonmetallicolous populations accumulate zinc and cadmium more effectively than those from metallicolous populations?. New Phytol.

[CR11] Blažević I, Montaut S, Burčul F (2020). Glucosinolate structural diversity, identification, chemical synthesis and metabolism in plants. Phytochemistry.

[CR12] Boyd RS (2007). The defense hypothesis of elemental hyperaccumulation: status, challenges and new directions. Plant Soil.

[CR13] Boyd RS (2012). Plant defense using toxic inorganic ions: conceptual models of the defensive enhancement and joint effects hypotheses. Plant Sci.

[CR14] Boyd RS (2013). Exploring tradeoffs in hyperaccumulator ecology and evolution. New Phytol.

[CR15] Broadhurst CL, Chaney RL, Angle JS (2004). Simultaneous hyperaccumulation of nickel, manganese, and calcium in *Alyssum* leaf trichomes. Environ Sci Technol.

[CR16] Christensen S, Enge S, Jensen KR (2019). Different herbivore responses to two co-occurring chemotypes of the wild crucifer *Barbarea vulgaris*. Arthropod Plant Interact.

[CR17] Clemens S (2001). Molecular mechanisms of plant metal tolerance and homeostasis. Planta.

[CR18] Dalin P, Agren J, Bjorkman C, Schaller A (2008). Leaf trichome formation and plant resistance to herbivory Chap. 4. Induced plant resistance to herbivory.

[CR19] Deshmukh R, Sonah H, Belanger RR (2020). New evidence defining the evolutionary path of aquaporins regulating silicon uptake in land plants. J Exp Bot.

[CR20] Detmann KC, Araujo WL, Martins SC (2012). Silicon nutrition increases grain yield, which, in turn, exerts a feed-forward stimulation of photosynthetic rates via enhanced mesophyll conductance and alters primary metabolism in rice. New Phytol.

[CR21] Epstein E (1999). Silicon. Annu Rev Plant Physiol Plant Mol Biol.

[CR22] Ernst WHO, Krauss GJ, Verkleij JAC, Wesenberg D (2008). Interaction of heavy metals with the sulphur metabolism in angiosperms from an ecological point of view. Plant Cell Environ.

[CR23] FAO, UNEP (2021). Global assessment of soil pollution: Report.

[CR24] Fones HN, Preston GM (2013). Trade-offs between metal hyperaccumulation and induced Disease resistance in metal hyperaccumulator plants. Plant Pathol.

[CR25] Fones HN, Preston GM, Smith JAC (2019). Variation in defence strategies in the metal hyperaccumulator plant Noccaea caerulescens is indicative of synergies and trade-offs between forms of defence. R Soc Open Sci.

[CR26] Fox J, Weisberg S (2019) An R companion to applied regression. Third edition. Sage, Thousand Oaks CA. https://socialsciences.mcmaster.ca/jfox/Books/Companion/

[CR27] Freeman JL, Persans MW, Nieman K (2004). Increased glutathione biosynthesis plays a role in nickel tolerance in *Thlaspi* nickel hyperaccumulators. Plant Cell.

[CR28] Fukuda N, Hokura A, Kitajima N (2008). Micro X-ray fluorescence imaging and micro X-ray absorption spectroscopy of cadmium hyper-accumulating plant, Arabidopsis halleri ssp. gemmifera, using high-energy synchrotron radiation. J Anal At Spectrom.

[CR29] Gols R, Wagenaar R, Bukovinszky T (2008). Genetic variation in defense chemistry in wild cabbages affects herbivores and their endoparasitoids. Ecology.

[CR30] Grill E, Loffler S, Winnacker E-L, Zenk MH (1989). Phytochelatins, the heavy-metal-binding peptides of plants, are synthesized from glutathione by a specific γ-glutamylcysteine dipeptidyl transpeptidase (phytochelatin synthase). Proc Natl Acad Sci.

[CR31] Hall CR, Waterman JM, Vandegeer RK (2019). The role of silicon in antiherbivore phytohormonal signalling. Front Plant Sci.

[CR32] Harrison S, Rajakaruna N (2011). Serpentine: the evolution and ecology of a model system.

[CR33] Honjo MN, Kudoh H (2019). *Arabidopsis halleri*: a perennial model system for studying population differentiation and local adaptation. AoB Plants.

[CR34] Hopkins RJ, Van Dam NM, Van Loon JJA (2009). Role of glucosinolates in insect-plant relationships and multitrophic interactions. Annu Rev Entomol.

[CR35] Hothorn T, Bretz F, Westfall P et al (2021) Package ‘multcomp’

[CR36] Kaiser BN, Gridley KL, Brady JN (2005). The role of molybdenum in agricultural plant production. Ann Bot.

[CR37] Karabourniotis G, Liakopoulos G, Nikolopoulos D, Bresta P (2020). Protective and defensive roles of non-glandular trichomes against multiple stresses: structure–function coordination. J For Res.

[CR38] Kassambara A (2023) ggpubr: ‘ggplot2’ Based Publication Ready Plots. R package version 0.6.0, https://rpkgs.datanovia.com/ggpubr/

[CR39] Kazemi-Dinan A, Thomaschky S, Stein RJ (2014). Zinc and cadmium hyperaccumulation act as deterrents towards specialist herbivores and impede the performance of a generalist herbivore. New Phytol.

[CR40] Kazemi-Dinan A, Barwinski A, Stein RJ (2015). Metal hyperaccumulation in Brassicaceae mediates defense against herbivores in the field and improves growth. Entomol Exp Appl.

[CR41] Kazemi-Dinan A, Sauer J, Stein RJ (2015). Is there a trade-off between glucosinolate-based organic and inorganic defences in a metal hyperaccumulator in the field?. Oecologia.

[CR42] Khan I, Awan SA, Rizwan M (2021). Effects of silicon on heavy metal uptake at the soil-plant interphase: a review. Ecotoxicol Environ Saf.

[CR43] Kim YH, Khan AL, Kim DH (2014). Silicon mitigates heavy metal stress by regulating P-type heavy metal ATPases, Oryza sativa low silicon genes, and endogenous phytohormones. BMC Plant Biol.

[CR44] Krämer U (2010). Metal hyperaccumulation in plants. Annu Rev Plant Biol.

[CR45] Krämer U, Grime GW, Smith JAC (1997). Micro-PIXE as a technique for studying nickel localization in leaves of the hyperaccumulator plant *Alyssum lesbiacum*. Nucl Instrum Methods Phys Res Sect B Beam Interact Mater Atoms.

[CR46] Kumar S, Soukup M, Elbaum R (2017). Silicification in grasses: variation between different cell types. Front Plant Sci.

[CR47] Liu WS, Zheng HX, Liu C (2021). Variation in rare earth element (REE), aluminium (Al) and silicon (Si) accumulation among populations of the hyperaccumulator *Dicranopteris linearis* in southern China. Plant Soil.

[CR48] Lu SG, Tang C, Rengel Z (2004). Combined effects of waterlogging and salinity on electrochemistry, water-soluble cations and water dispersible clay in soils with various salinity levels. Plant Soil.

[CR49] Meena V, Dotaniya ML, Saha JK, Patra AK (2021). Silicon potential to mitigate plant heavy metals stress for sustainable agriculture: a review. Silicon.

[CR50] Moore BD, Andrew RL, Külheim C, Foley WJ (2014). Explaining intraspecific diversity in plant secondary metabolites in an ecological context. New Phytol.

[CR51] Müller C, Junker RR (2022). Chemical phenotype as important and dynamic niche dimension of plants. New Phytol.

[CR52] Nakamura R, Amada G, Kajino H (2022). Silicious trichomes as a trait that may slow down leaf decomposition by soil meso- and macrofauna. Plant Soil.

[CR53] Neumann D, Zur Nieden U (2001). Silicon and heavy metal tolerance of higher plants. Phytochemistry.

[CR54] Neumann D, De Figueiredo C (2002). A novel mechanism of silicon uptake. Protoplasma.

[CR55] Ofoe R, Thomas RH, Asiedu SK (2023). Aluminum in plant: benefits, toxicity and tolerance mechanisms. Front Plant Sci.

[CR56] Oksanen J, Blanchet FG, Kindt R et al. (2020) Package “vegan”: community ecology package. R Packag. version 2.3-1 264

[CR57] Pongrac P, Tolrà R, Vogel-Mikuš K et al (2010) At the crossroads of metal hyperaccumulation and glucosinolates: Is there anything out there? In: Soil Heavy Metals. pp 139–161. Soil Biology, vol 19. Springer, Berlin, Heidelberg. 10.1007/978-3-642-02436-8_7

[CR58] Putra R, Müller C (2023). Extending the elemental defence hypothesis in the light of plant chemodiversity. New Phytol.

[CR59] Putra R, Powell JR, Hartley SE, Johnson SN (2020). Is it time to include legumes in plant silicon research?. Funct Ecol.

[CR60] Putra R, Vandegeer RK, Karan S (2021). Silicon enrichment alters functional traits in legumes depending on plant genotype and symbiosis with nitrogen-fixing bacteria. Funct Ecol.

[CR61] R Core Team (2021) R: A language and environment for statistical computing

[CR62] Rausch T, Wachter A (2005). Sulfur metabolism: a versatile platform for launching defence operations. Trends Plant Sci.

[CR63] Reeves RD, Baker AJM, Jaffré T (2018). A global database for plants that hyperaccumulate metal and metalloid trace elements. New Phytol.

[CR64] Rieuwerts JS, Thornton I, Farago ME, Ashmore MR (1998). Factors influencing metal bioavailability in soils: preliminary investigations for the development of a critical loads approach for metals. Chem Speciat Bioavailab.

[CR65] Rylott EL, Bruce NC (2022). Plants to mine metals and remediate land. Science.

[CR66] Schmitt M, Watanabe T, Jansen S (2016). The effects of aluminium on plant growth in a temperate and deciduous aluminium accumulating species. AoB Plants.

[CR67] Stein RJ, Höreth S, de Melo JRF (2017). Relationships between soil and leaf mineral composition are element-specific, environment-dependent and geographically structured in the emerging model *Arabidopsis halleri*. New Phytol.

[CR68] Stolpe C, Krämer U, Müller C (2017). Heavy metal (hyper)accumulation in leaves of *Arabidopsis Halleri* is accompanied by a reduced performance of herbivores and shifts in leaf glucosinolate and element concentrations. Environ Exp Bot.

[CR69] Tang Y, Horikoshi M, Li W (2016). Ggfortify: unified interface to visualize statistical result of popular R packages. R J.

[CR70] Tewes LJ, Müller C (2020). Interactions of *Bunias orientalis* plant chemotypes and fungal pathogens with different host specificity in vivo and in vitro. Sci Rep.

[CR71] Tewes LJ, Stolpe C, Kerim A (2018). Metal hyperaccumulation in the brassicaceae species *Arabidopsis Halleri* reduces camalexin induction after fungal pathogen attack. Environ Exp Bot.

[CR72] Textor S, Gershenzon J (2009). Herbivore induction of the glucosinolate-myrosinase defense system: major trends, biochemical bases and ecological significance. Phytochem Rev.

[CR73] Tolrà RP, Poschenrieder C, Alonso R (2001). Influence of zinc hyperaccumulation on glucosinolates in *Thlaspi caerulescens*. New Phytol.

[CR74] Tolrà R, Pongrac P, Poschenrieder C (2006). Distinctive effects of cadmium on glucosinolate profiles in cd hyperaccumulator *Thlaspi praecox* and non-hyperaccumulator *Thlaspi arvense*. Plant Soil.

[CR75] Van De Mortel JE, Schat H, Moerland PD (2008). Expression differences for genes involved in lignin, glutathione and sulphate metabolism in response to cadmium in *Arabidopsis thaliana* and the related Zn/Cd-hyperaccumulator *Thlaspi caerulescens*. Plant Cell Environ.

[CR76] Violante A, Cozzolino V, Perelomov L (2010). Mobility and bioavailability of heavy metals and metalloids in soil environments. J Soil Sci Plant Nutr.

[CR77] Wang M, Zheng Q, Shen Q, Guo S (2013). The critical role of potassium in plant stress response. Int J Mol Sci.

[CR78] Wickham H (2016). ggplot2: elegant graphics for data analysis.

[CR79] Wickham H, Hester J, Chang W (2021) Package ‘ devtools ’

[CR80] Wierzbicka M, Pielichowska M, Abratowska A (2016). Thallium hyperaccumulation in polish populations of *Biscutella laevigata* (Brassicaceae). Acta Biol Cracoviensia Ser Bot.

[CR81] Xu D, Gao T, Fang X (2020). Silicon addition improves plant productivity and soil nutrient availability without changing the grass:legume ratio response to N fertilization. Sci Rep.

[CR82] Yan A, Pan J, An L (2012). The responses of trichome mutants to enhanced ultraviolet-B radiation in *Arabidopsis thaliana*. J Photochem Photobiol B Biol.

[CR83] Zhao FJ, Lombi E, Breedon T, McGrath SP (2000). Zinc hyperaccumulation and cellular distribution in *Arabidopsis halleri*. Plant Cell Environ.

[CR84] Zheng HX, Yang YL, Liu WS (2023). Rare earth elements detoxification mechanism in the hyperaccumulator *Dicranopteris linearis*: [silicon-pectin] matrix fixation. J Hazard Mater.

