# Supplementary material for: Effects of metal amendment and metalloid supplementation on foliar defences are plant accession-specific in the hyperaccumulator Arabidopsis halleri
Source: Biometals. 2023 Oct 24;37(3):649–69. doi: 10.1007/s10534-023-00550-5 (PMC11101560; doi:10.1007/s10534-023-00550-5)
Supplement: Supplementary file 1 — Supplementary material 1 (DOCX 1057.2 kb) [file 10534_2023_550_MOESM1_ESM.docx]

**Effects of metal amendment and metalloid supplementation on foliar defences are plant accession-specific in the hyperaccumulator *Arabidopsis halleri***

**Supplementary information**


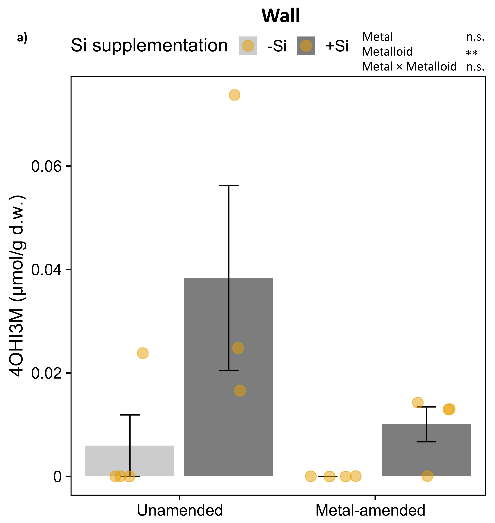

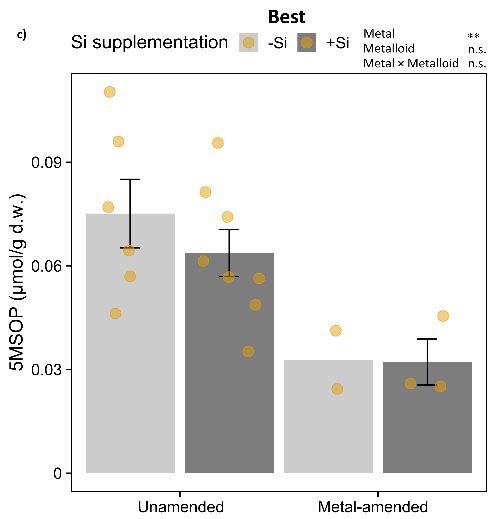

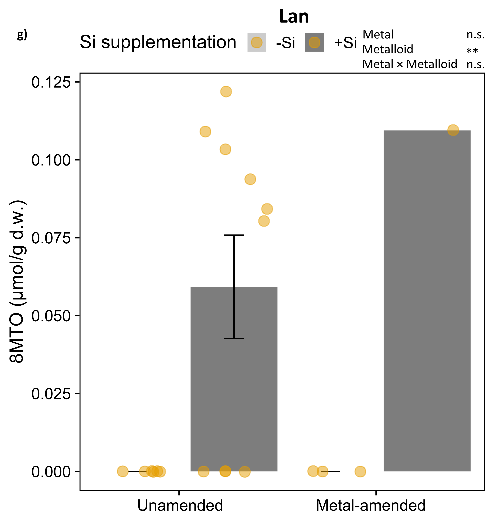


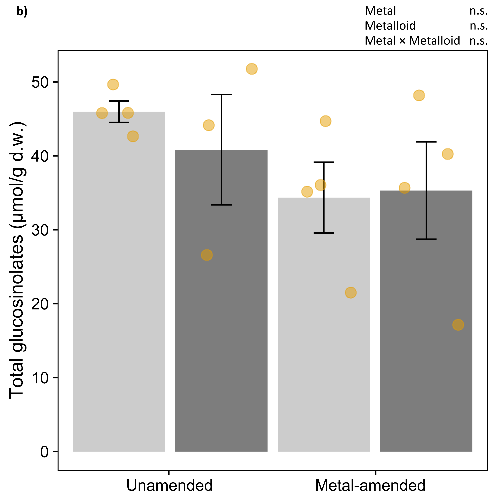

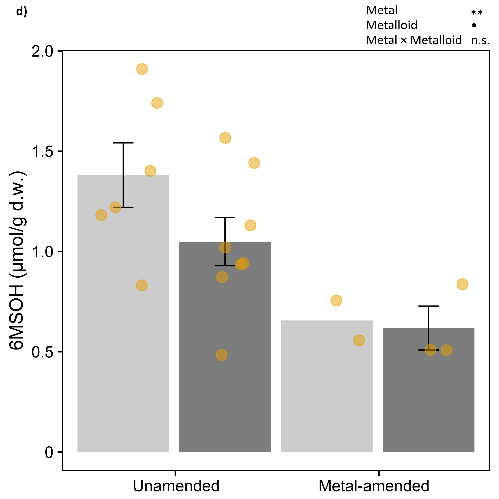

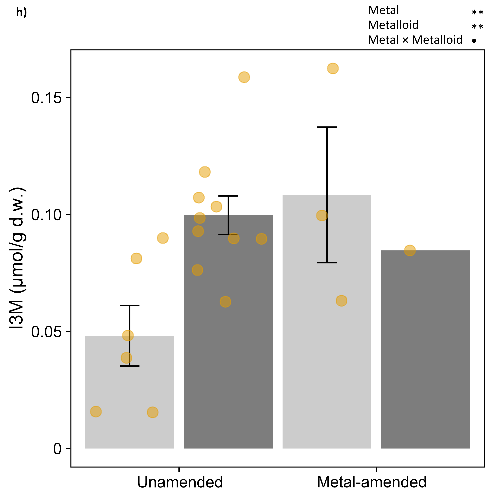


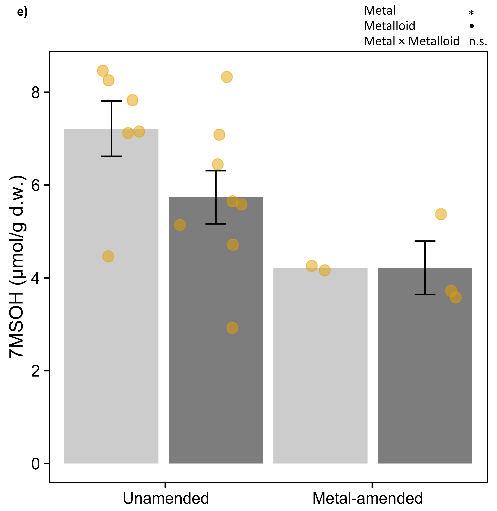

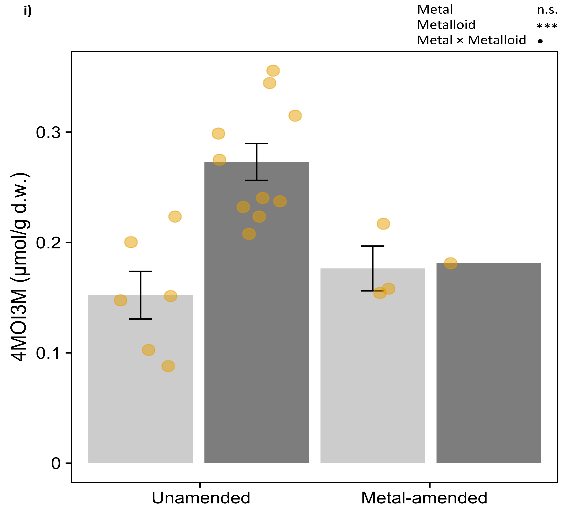


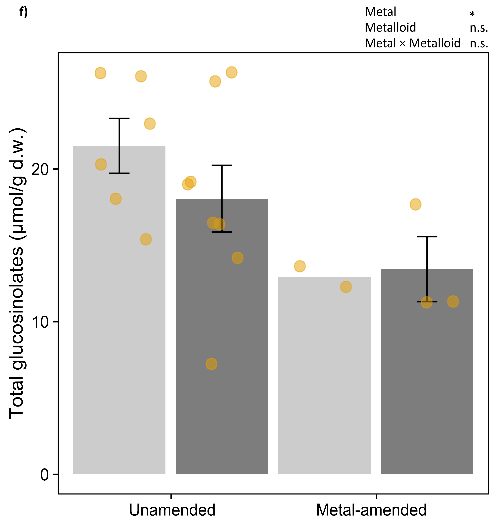

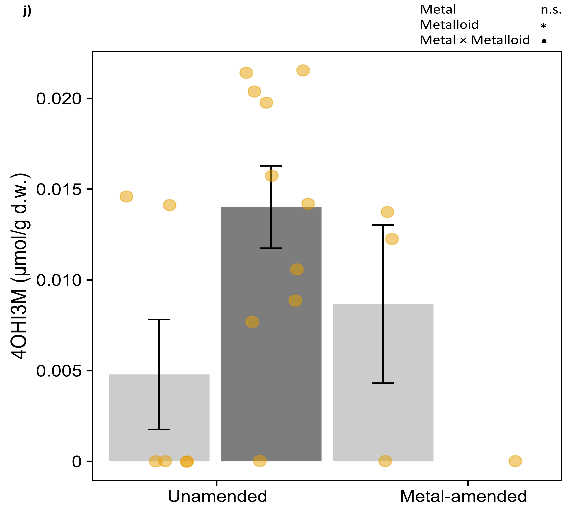


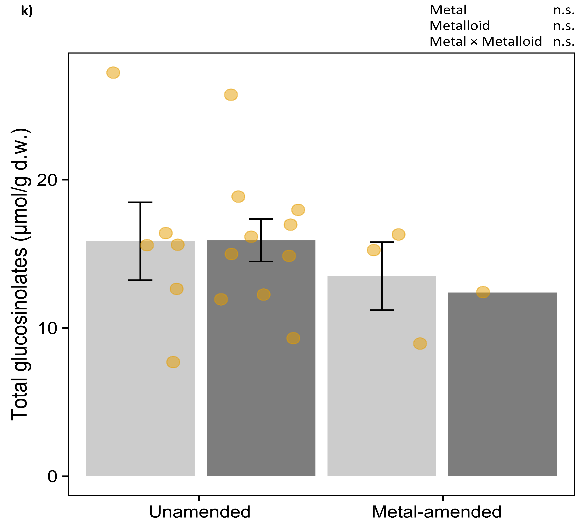


**Fig. S1** Foliar concentrations (mean ± SE in μmol/g d.w.) of selected and total glucosinolates in plants of three accessions of *Arabidopsis halleri* (Wall: Wallenfels, Best: Bestwig and Lan: Langelsheim) grown on soil without or with metal amendment and metalloid (Si) supplementation. Solid circles indicate data points: *n* = 3–10 per treatment combination, except *n* = 2 for the Best accession with metal amendment and -Si supplementation and *n* = 1 for the Lan accession with metal amendment and +Si supplementation. Statistical outcomes are indicated as: ******* *P* < 0.001, ****** *P* < 0.01, ***** *P* < 0.05, **•** *P* < 0.1 (marginally significant) and n.s. *P* > 0.1 (non-significant). Different letters above the bars indicate significant differences based on the Tukey’s HSD post-hoc test. Glucosinolates are abbreviated as: 5MSOP: 5-methylsulfinylpentyl glucosinolate; 6MSOH: 6-methylsulfinylhexyl glucosinolate; 7MSOH: 7-methylsulfinylheptyl glucosinolate; 8MTO: 8-methylthiooctyl glucosinolate; I3M: indol-3-ylmethyl glucosinolate; 4MOI3M: 4-methoxyindol-3-ylmethyl glucosinolate and 4OHI3M: 4-hydroxyindol-3-ylmethyl glucosinolate.


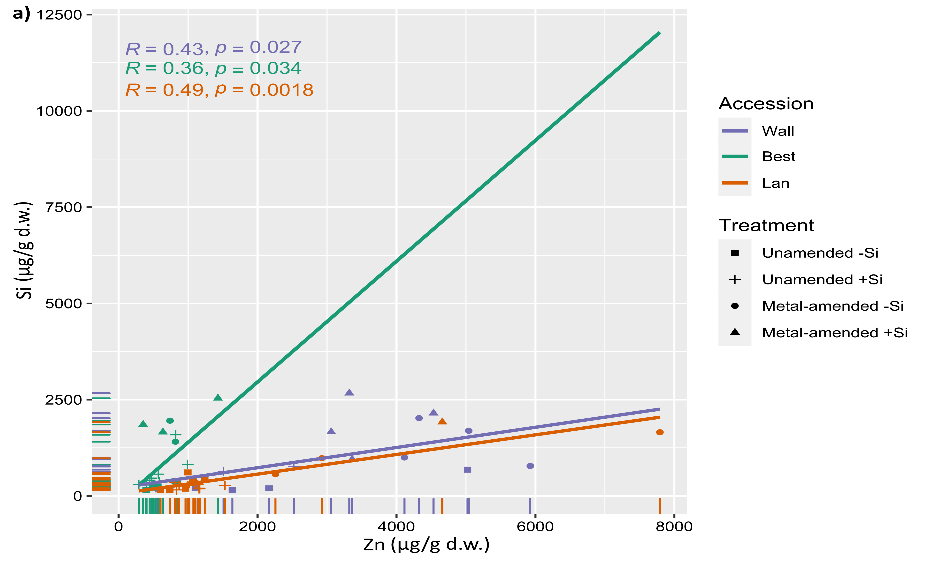

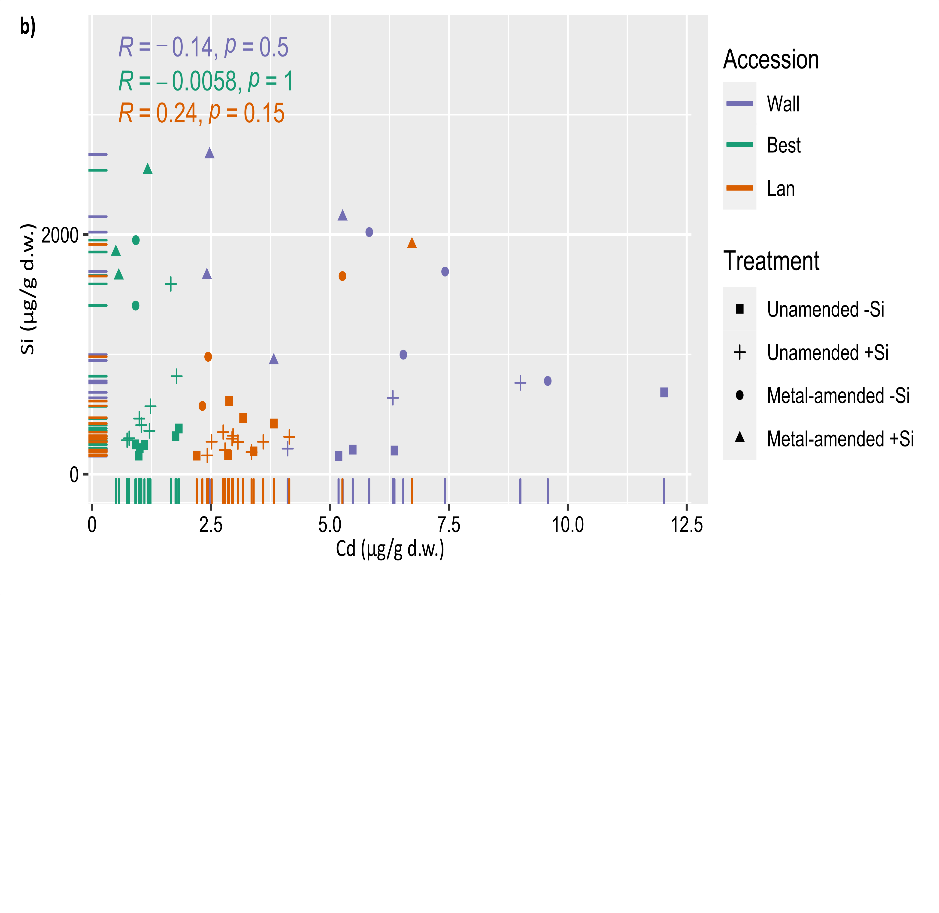

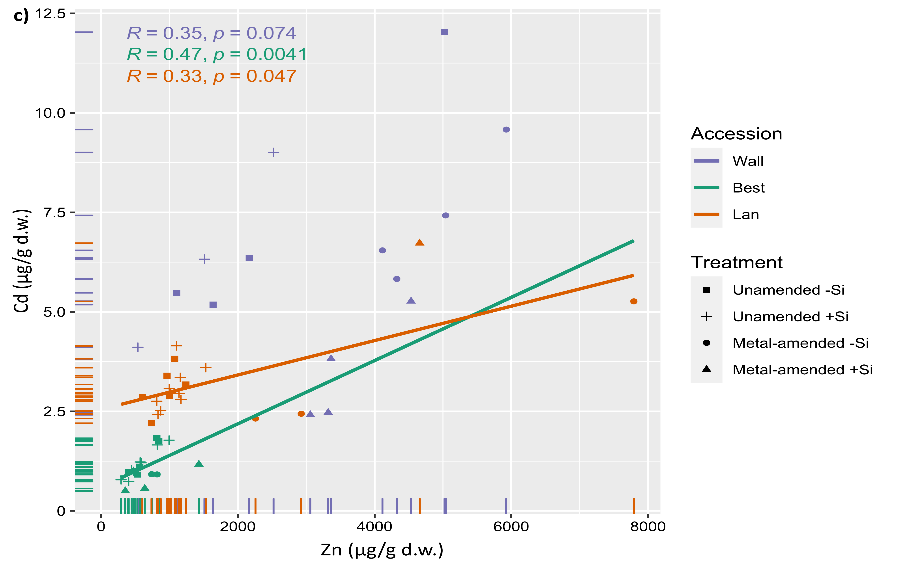

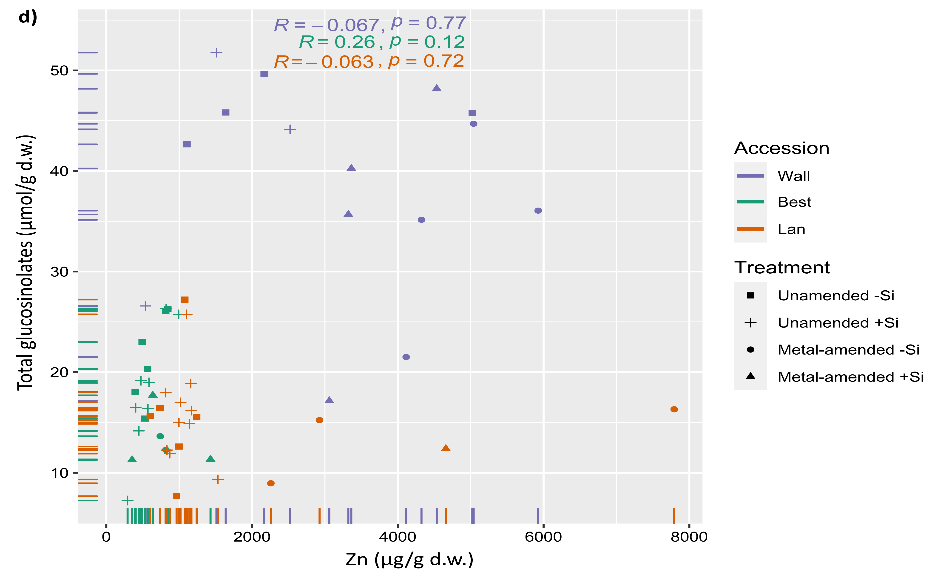

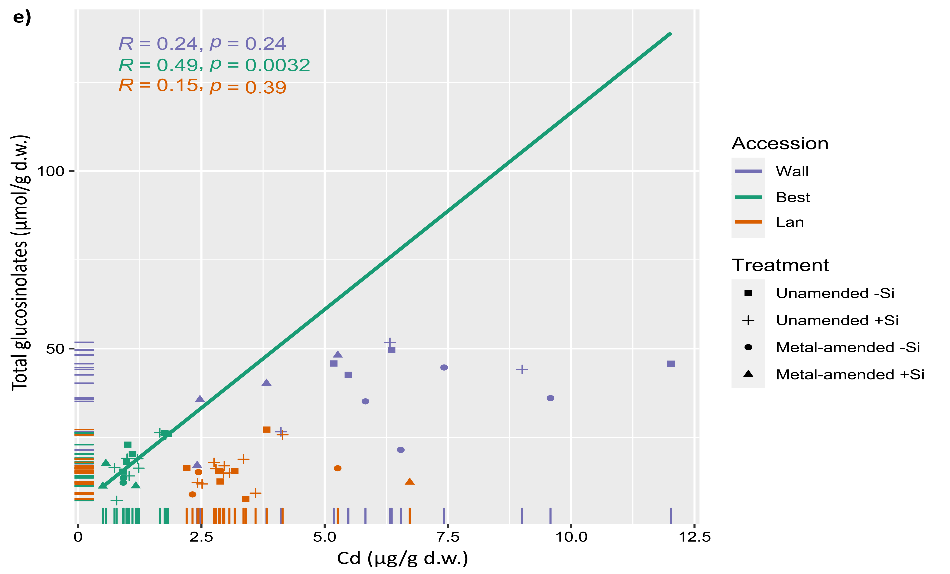

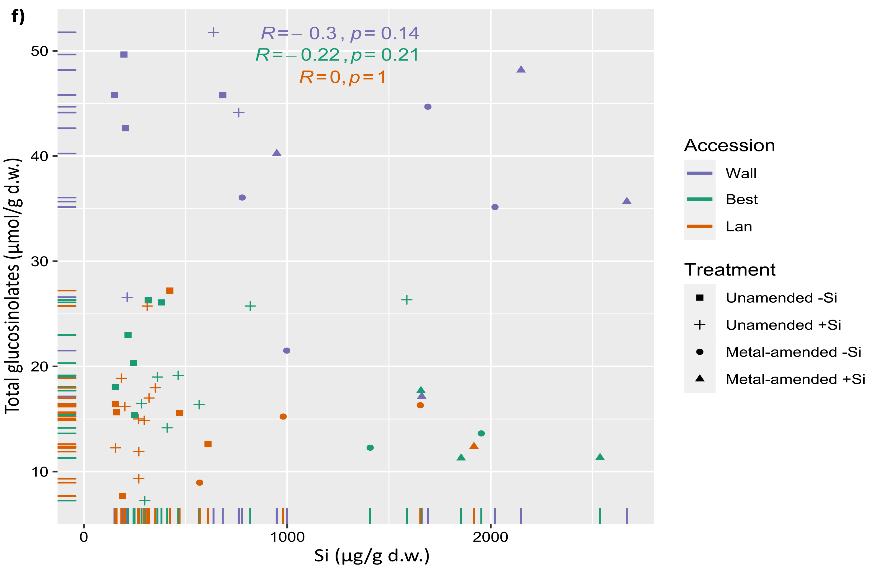


**Fig. S2** Correlations between concentrations of Cd, Zn, Si and total glucosinolates in leaves of three accessions of *Arabidopsis halleri* (Wall: Wallenfels, Best: Bestwig and Lan: Langelsheim) grown on soil without or with metal amendment and metalloid (Si) supplementation. Significant correlations with *P*-value < 0.05 are indicated by solid lines.

**Table S1** Effects of metal amendment, metalloid (Si) supplementation and their interactions on foliar concentrations of elements (μg/g d.w.) in the three accessions of *Arabidopsis halleri* (Wall: Wallenfels, Best: Bestwig and Lan: Langelsheim) based on a generalised linear model with a Gaussian distribution. *n* = 4–10 per treatment combination, except *n* = 2 for the Lan accession with metal amendment and +Si supplementation. Significant *P*-values (*P* < 0.05) are highlighted in bold.

| Elements per accession | Factor | | | | | | | | |
| --- | --- | --- | --- | --- | --- | --- | --- | --- | --- |
|  | Metal | | | Metalloid | | | Metal × Metalloid | | |
|  | *df* | *χ^2^* | *P* | *df* | *χ^2^* | *P* | *df* | *χ^2^* | *P* |
| **Wall** | | | | | | | | | |
| B | 1 | 0.96 | 0.33 | 1 | 0.1 | 0.75 | 1 | 0.12 | 0.73 |
| Ca | 1 | 0.91 | 0.34 | 1 | 0.19 | 0.66 | 1 | 0.0004 | 0.98 |
| Cu | 1 | 2.14 | 0.14 | 1 | 0.08 | 0.77 | 1 | 0.14 | 0.71 |
| Mg | 1 | 0.81 | 0.37 | 1 | 1.01 | 0.31 | 1 | 0.01 | 0.94 |
| Mo | 1 | 19.11 | **< 0.001** | 1 | 0.07 | 0.8 | 1 | 1.43 | 0.23 |
| P | 1 | 19.25 | **< 0.001** | 1 | 0.33 | 0.57 | 1 | 2.79 | 0.09 |
| **Best** | | | | | | | | | |
| B | 1 | 0.32 | 0.57 | 1 | 0.27 | 0.6 | 1 | 1.49 | 0.22 |
| Ca | 1 | 0.37 | 0.54 | 1 | 0.03 | 0.85 | 1 | 3.23 | 0.07 |
| Cu | 1 | 0.2 | 0.66 | 1 | 0.64 | 0.42 | 1 | 2.99 | 0.08 |
| Mg | 1 | 0.15 | 0.7 | 1 | 0.07 | 0.79 | 1 | 0.03 | 0.87 |
| Mo | 1 | 0.24 | 0.62 | 1 | 0.25 | 0.62 | 1 | 0.97 | 0.33 |
| P | 1 | 2.37 | 0.12 | 1 | 1.32 | 0.25 | 1 | 0.89 | 0.35 |
| **Lan** | | | | | | | | | |
| B | 1 | 11.99 | **< 0.001** | 1 | 0.37 | 0.54 | 1 | 0.63 | 0.43 |
| Ca | 1 | 0.33 | 0.56 | 1 | 3.34 | 0.07 | 1 | 3.76 | 0.052 |
| Cu | 1 | 0.03 | 0.86 | 1 | 2.56 | 0.11 | 1 | 1.25 | 0.26 |
| Mg | 1 | 1.68 | 0.2 | 1 | 1.72 | 0.19 | 1 | 0.19 | 0.66 |
| Mo | 1 | 26.35 | **< 0.001** | 1 | 0.0001 | 0.99 | 1 | 0.002 | 0.96 |
| P | 1 | 16.7 | **< 0.001** | 1 | 0.08 | 0.78 | 1 | 0.07 | 0.79 |
